# Assimilatory sulfate reduction in the marine methanogen *Methanothermococcus thermolithotrophicus*

**DOI:** 10.1038/s41564-023-01398-8

**Published:** 2023-06-05

**Authors:** Marion Jespersen, Tristan Wagner

**Affiliations:** Microbial Metabolism Group, Max Planck Institute for Marine Microbiology, Bremen, Germany

**Keywords:** Archaea, Enzymes, X-ray crystallography

## Abstract

*Methanothermococcus thermolithotrophicus* is the only known methanogen that grows on sulfate as its sole sulfur source, uniquely uniting methanogenesis and sulfate reduction. Here we use physiological, biochemical and structural analyses to provide a snapshot of the complete sulfate reduction pathway of this methanogenic archaeon. We find that later steps in this pathway are catalysed by atypical enzymes. PAPS (3′-phosphoadenosine 5′-phosphosulfate) released by APS kinase is converted into sulfite and 3′-phosphoadenosine 5′-phosphate (PAP) by a PAPS reductase that is similar to the APS reductases of dissimilatory sulfate reduction. A non-canonical PAP phosphatase then hydrolyses PAP. Finally, the F_420_-dependent sulfite reductase converts sulfite to sulfide for cellular assimilation. While metagenomic and metatranscriptomic studies suggest that the sulfate reduction pathway is present in several methanogens, the sulfate assimilation pathway in *M. thermolithotrophicus* is distinct. We propose that this pathway was ‘mix-and-matched’ through the acquisition of assimilatory and dissimilatory enzymes from other microorganisms and then repurposed to fill a unique metabolic role.

## Abstract (web summary)

Structural and biochemical analyses show how one archaeon links both sulfur reduction and methane production.

## Main

The most common methane-producing microorganisms have a high demand for sulfur due to their specific enzymes and metabolism. While most of these methanogens use sulfides (HS^−^), some have been shown to metabolize higher oxidation states of sulfur or even metal sulfides (for example, FeS_2_) for sulfur acquisition^1–5^. However, *Methanothermococcus thermolithotrophicus* is the only known methanogen capable of growing on sulfate (${{\rm{SO}}}_{4}^{2-}$) as its sole sulfur source^4,6^. The metabolism of this marine hydrogenotroph, isolated from geothermally heated sea sediments near Naples (Italy), is paradoxical, as ${{\rm{SO}}}_{4}^{2-}$ reduction should lead to several physiological obstacles for a methane-producing microbe. First, methanogens commonly thrive in reduced sulfidic environments where all electron acceptors other than CO_2_ are depleted, including ${{\rm{SO}}}_{4}^{2-}$ (refs. ^7,8^). Second, at the interface where methanogens and ${{\rm{SO}}}_{4}^{2-}$ ions coexist, hydrogenotrophic methanogens must compete with dissimilatory ${{\rm{SO}}}_{4}^{2-}$-reducing microorganisms for the common substrate dihydrogen (H_2_)^9^. Third, methanogens live at the thermodynamic limits of life and the adenosine triphosphate (ATP) hydrolysis coupled to ${{\rm{SO}}}_{4}^{2-}$ reduction would be a substantial investment for such energy-limited microorganisms^8,10^. Finally, the ${{\rm{SO}}}_{4}^{2-}$ reduction pathway generates toxic intermediates that would interfere with cellular processes.

**Extended Data Fig. 1:**
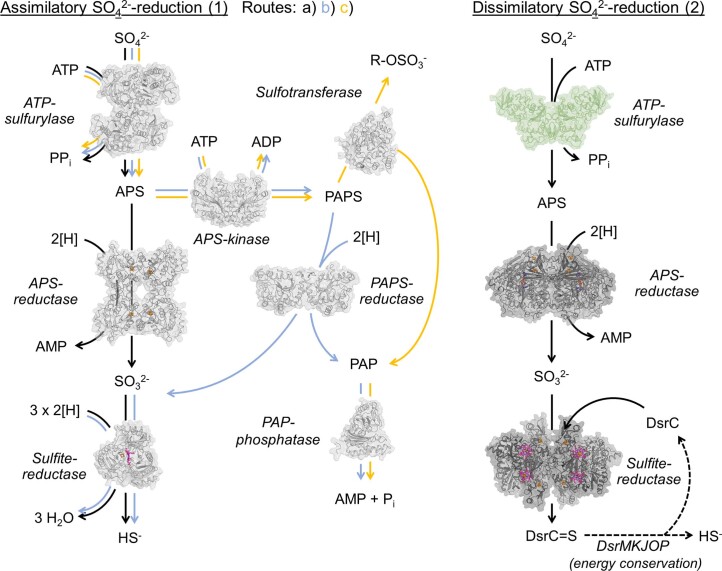
Sulfate (${\mathbf{SO}}_{\mathbf{4}}^{{\mathbf{2-}}}$)-reduction pathways. Assimilatory ${{\rm{SO}}}_{4}^{2-}$-reduction. (Route 1a, 1b, 1c) ${{\rm{SO}}}_{4}^{2-}$ is activated by the ATP-sulfurylase (for example *Glycine max*, PDB: 4MAF) to APS. (1a) APS gets directly reduced to sulfite (${{\rm{SO}}}_{3}^{2-}$) and AMP by a one [4Fe-4S]-cluster containing APS-reductase (for example *Pseudomonas aeruginosa*, PDB: 2GOY, thioredoxin dependent). (1b, c) Alternatively, the APS gets further phosphorylated by an APS-kinase (for example *Arabidopsis thaliana*, PDB: 3UIE) to produce PAPS. (1b) A PAPS-reductase (*Saccharomyces cerevisiae*, PDB: 2OQ2, thioredoxin dependent) converts PAPS into ${{\rm{SO}}}_{3}^{2-}$ and PAP. The PAP will be hydrolysed to inorganic phosphate (P_i_) and AMP by a PAP-phosphatase (for example *Mycobacterium tuberculosis*, PDB: 5DJJ). (1a, b) A sulfite-reductase (*Escherichia coli*, PDB: 1AOP) reduces the ${{\rm{SO}}}_{3}^{2-}$ into S^2−^, which can then be incorporated into biomass. In the route 1c, a sulfotransferase (for example *A. thaliana*, PDB: 5MEK) catalyses the transfer of the sulfo-group (R-OSO_3_^−^) from PAPS to an alcohol or amine acceptor. Dissimilatory ${{\rm{SO}}}_{4}^{2-}$-reduction. ${{\rm{SO}}}_{4}^{2-}$ is activated by the ATPS to APS and further reduced to ${{\rm{SO}}}_{3}^{2-}$ by an APS-reductase (for example *Archaeoglobus fulgidus*, PDB: 2FJA), which contains two [4Fe-4S]-cluster and a FAD. A sulfite-reductase (for example *A. fulgidus*, PDB: 3MM5) reduces the ${{\rm{SO}}}_{3}^{2-}$ and branches it on the carrier DsrC. The membrane complex DsrMKJOP (for example *Allochromatium vinosum*) reduces the sulfur into HS^−^ concomitantly with ion translocation for energy conservation. Sirohemes, FAD and [4Fe-4S]-clusters are represented in sticks and spheres, with carbon, oxygen, nitrogen, sulfur and iron coloured in pink, red, blue, yellow and orange. The enzymes are shown in cartoon and transparent surface in their oligomeric state. The ATPS of *A. fulgidus* was modelled using Alphafold2^32^ and coloured in green. The bifunctional ATP-sulfurylase CysDN using an additional GTP, was not presented here to simplify the scheme.

To assimilate ${{\rm{SO}}}_{4}^{2-}$, the organism would have to capture the anion and transport it into the cell using a transporter. Inside the cell, ${{\rm{SO}}}_{4}^{2-}$ is activated by an ATP sulfurylase (ATPS) to generate adenosine 5′-phosphosulfate (APS)^11–13^. From there, organisms can use different strategies (Extended Data Fig. 1, routes a–c): (1a) APS is directly reduced by an APS reductase (APSR) to generate AMP and ${{\rm{SO}}}_{3}^{2-}$. (1b) Alternatively, APS can be further phosphorylated to 3′-phosphoadenosine 5′-phosphosulfate (PAPS) by the APS kinase (APSK). A PAPS reductase (PAPSR) will reduce PAPS to ${{\rm{SO}}}_{3}^{2-}$ and the toxic nucleotide 3’-phosphoadenosine 5′-phosphate (PAP). PAP must be quickly hydrolysed to AMP and inorganic phosphate by a PAP phosphatase (PAPP). In both scenarios, the final step is carried out by a siroheme-containing sulfite reductase, which reduces the ${{\rm{SO}}}_{3}^{2-}$ into HS^−^. The latter can then be incorporated into biomass. (1c) In a different pathway, the sulfite group of PAPS is transferred to another acceptor to build up sulfated metabolites. Route 1a is very similar to the dissimilatory pathway (Extended Data Fig. 1, route 2). However, dissimilatory APSRs and dissimilatory sulfite reductases are structurally and phylogenetically distinct from their assimilatory counterparts and indirectly couple their reactions to membrane pumps, allowing for energy conservation^14–16^.

Genes encoding putative enzymes associated with ${{\rm{SO}}}_{4}^{2-}$ reduction have been found in the genomes of multiple methanogens^13^, including *M. thermolithotrophicus*. For this methanogen, a theoretical, albeit incomplete, ${{\rm{SO}}}_{4}^{2-}$ assimilation pathway can be hypothesized. Here we elucidated the complete ${{\rm{SO}}}_{4}^{2-}$ reduction machinery of this archaeon and describe how this one methanogen can convert ${{\rm{SO}}}_{4}^{2-}$ into an elementary block of life.

## Results

### A marine methanogen consuming ${\mathbf{SO}}_{\mathbf{4}}^{\mathbf{2-}}$

**Fig. 1:**
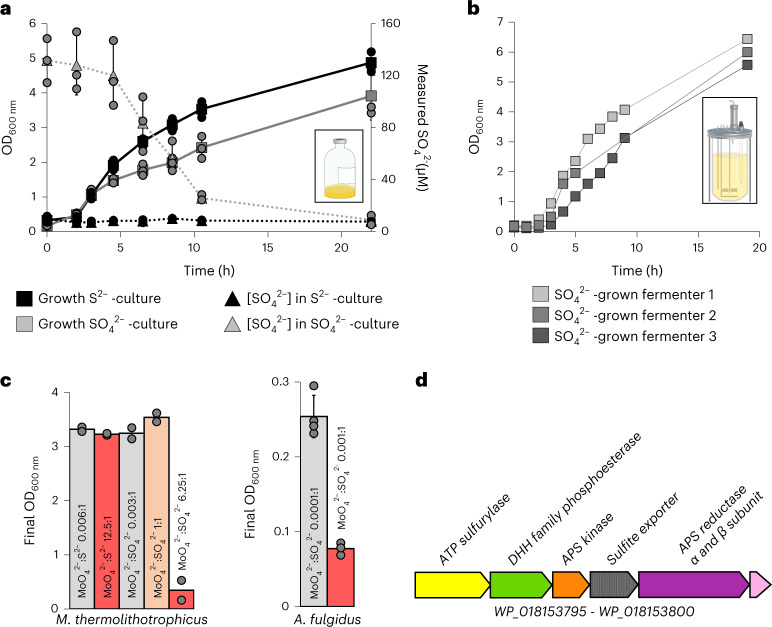
${\mathbf{SO}}_{\mathbf{4}}^{{\mathbf{2-}}}$ growth dependency of *M. thermolithotrophicus*. **a**, *M. thermolithotrophicus* cultures grown on Na_2_S (black squares, 0.5 mM) and Na_2_SO_4_ (grey squares, 0.5 mM). The consumption or release of ${{\rm{SO}}}_{4}^{2-}$ in Na_2_S or Na_2_SO_4_ cultures are shown by black and grey triangles, respectively. Data are presented as mean ± s.d. and individual values are shown as spheres (*n* = 3 replicates). Differences between expected (0.5 mM) and measured (0.13 mM) ${{\rm{SO}}}_{4}^{2-}$ concentration for the initial point are considered to be due to an artefact from the medium (see Methods). **b**, *M. thermolithotrophicus* grown on 10 mM Na_2_SO_4_ in three independent fermenters. The sampling points are represented by grey squares. **c**, Molybdate (Na_2_MoO_4_) inhibition of Na_2_SO_4_ assimilatory and dissimilatory archaea. Growth experiments for *M. thermolithotrophicus* were performed in duplicates and for *A. fulgidus* in quadruplicates (left) or triplicates (right). Data are represented as mean and for the triplicates and quadruplicates ± s.d. **d**, Predicted operon for ${{\rm{SO}}}_{4}^{2-}$ reduction from the whole-genome shotgun sequence of *M. thermolithotrophicus*. Source data

Cultures grown on Na_2_S were successively transferred to a sulfur-free medium until no growth was observed. *M. thermolithotrophicus* showed robust growth when at least 100 µM of Na_2_SO_4_ was supplemented in the medium and reached similar cell yields as the Na_2_S-grown culture. Under these cultivation conditions, ${{\rm{SO}}}_{4}^{2-}$ is consumed over time as cell density increases (Fig. 1a). When cells are grown only on Na_2_S, no ${{\rm{SO}}}_{4}^{2-}$ could be detected (Fig. 1a), indicating that *M. thermolithotrophicus* is not performing sulfide oxidation.

We then challenged the ${{\rm{SO}}}_{4}^{2-}$-grown culture by switching from batch to fermenter conditions, where H_2_S can escape to the gas phase and does not accumulate compared to flask conditions. In this open system with temperature and pH controlled, *M. thermolithotrophicus* grew to a maximum optical density (OD)_600nm_ of 6.45 within 19 h (Fig. 1b).

**Extended Data Fig. 2:**
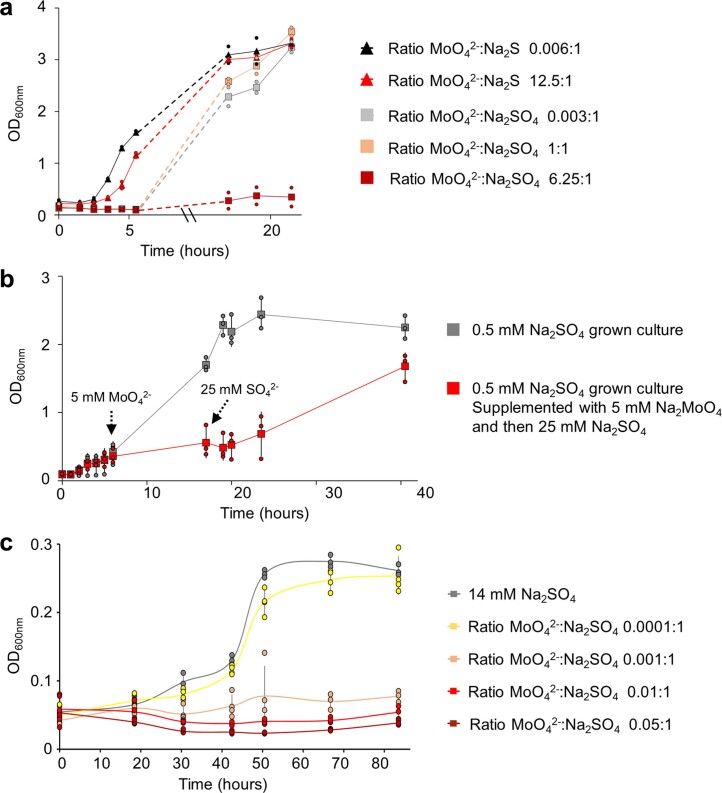
Impact of Molybdate (${\mathbf{MoO}}_{\mathbf{4}}^{{\mathbf{2-}}}$) on ${\mathbf{SO}}_{\mathbf{4}}^{{\mathbf{2-}}}$-reducing archaea. **a**, ${{\rm{MoO}}}_{4}^{2-}$ tolerance of *M. thermolithotrophicus*. The archaeon was grown on ${{\rm{SO}}}_{4}^{2-}$ (grey square), ${{\rm{SO}}}_{4}^{2-}$ supplemented with an equimolar amount of ${{\rm{MoO}}}_{4}^{2-}$ (wheat square) and ${{\rm{SO}}}_{4}^{2-}$ supplemented with an excess of ${{\rm{MoO}}}_{4}^{2-}$ (dark red square). As a control, Na_2_S grown cultures (S^2-^, black triangle) and Na_2_S grown cultures with an excess of ${{\rm{MoO}}}_{4}^{2-}$ (red triangle) were used. This growth experiment was performed in duplicates. **b**, Effect of ${{\rm{MoO}}}_{4}^{2-}$:${{\rm{SO}}}_{4}^{2-}$ ratios in *M. thermolithotrophicus* cultures grown on 0.5 mM Na_2_SO_4_. Grey squares indicate the growth curve of ${{\rm{SO}}}_{4}^{2-}$-reducers without addition of ${{\rm{MoO}}}_{4}^{2-}$. The red squares indicate the growth curve of ${{\rm{SO}}}_{4}^{2-}$ reducers exposed to 5 mM of ${{\rm{MoO}}}_{4}^{2-}$, followed by the addition of 25 mM Na_2_SO_4_. Black, dashed arrows indicate time of ${{\rm{MoO}}}_{4}^{2-}$ and ${{\rm{SO}}}_{4}^{2-}$ addition. This growth experiment was performed in triplicates. **c**, *Archaeoglobus fulgidus* sensitivity towards ${{\rm{MoO}}}_{4}^{2-}$. Here, a ${{\rm{MoO}}}_{4}^{2-}$:${{\rm{SO}}}_{4}^{2-}$ ratio of 0.001:1 is sufficient to inhibit growth of *A. fulgidus*. The data shown are quadruplicates except for the lowest and the highest ${{\rm{MoO}}}_{4}^{2-}$:${{\rm{SO}}}_{4}^{2-}$ ratio, which were performed in triplicates. All experiments are represented as data mean and for b, c ± standard deviation (s.d.). Source data

One way to determine whether *M. thermolithotrophicus* relies on canonical enzymes of the ${{\rm{SO}}}_{4}^{2-}$ reduction pathway is to use molybdate (${{\rm{MoO}}}_{4}^{2-}$). The structural analogue of ${{\rm{SO}}}_{4}^{2-}$ binds to the ATPS and triggers molybdolysis, which hydrolyses ATP to AMP and pyrophosphate (PP_i_), resulting in cellular energy depletion^17,18^. A ${{\rm{MoO}}}_{4}^{2-}$:${{\rm{SO}}}_{4}^{2-}$ molar ratio of 0.004:1 is sufficient to inhibit the activity of dissimilatory ${{\rm{SO}}}_{4}^{2-}$-reducing bacteria for 168 h, an effect mainly due to molybdolysis by ATPS^19–21^. ${{\rm{SO}}}_{4}^{2-}$ assimilation is also affected by ${{\rm{MoO}}}_{4}^{2-}$, as demonstrated by studies on plants^22^. In the latter, growth inhibition occurred when ${{\rm{MoO}}}_{4}^{2-}$ was in excess compared to ${{\rm{SO}}}_{4}^{2-}$ and the ATPS activity was notably affected at a 1:1 ratio^18^. When applied on *M. thermolithotrophicus*, a high ${{\rm{MoO}}}_{4}^{2-}$:Na_2_S ratio of 12.5:1 did not disturb growth of the Na_2_S culture, indicating that ${{\rm{MoO}}}_{4}^{2-}$ is not interfering with their basal metabolism. In contrast, a ${{\rm{MoO}}}_{4}^{2-}$:${{\rm{SO}}}_{4}^{2-}$ ratio of 6.25:1 was inhibitory to the ${{\rm{SO}}}_{4}^{2-}$-grown culture, while a 1:1 ratio was not (Fig. 1c and Extended Data Fig. 2a). ${{\rm{SO}}}_{4}^{2-}$ addition to the ${{\rm{MoO}}}_{4}^{2-}$-inhibited culture restored growth (Extended Data Fig. 2b), indicating the reversibility of inhibition and its strict control by the ${{\rm{MoO}}}_{4}^{2-}$:${{\rm{SO}}}_{4}^{2-}$ ratio rather than the ${{\rm{MoO}}}_{4}^{2-}$ concentration. In comparison, in *Archaeoglobus fulgidus*, an archaeon that performs dissimilatory ${{\rm{SO}}}_{4}^{2-}$ reduction to conserve energy, we observed growth inhibition at a ${{\rm{MoO}}}_{4}^{2-}$:${{\rm{SO}}}_{4}^{2-}$ ratio of 0.001:1 (Fig. 1c and Extended Data Fig. 2c). These results suggest that *M. thermolithotrophicus* reduces ${{\rm{SO}}}_{4}^{2-}$ via an assimilatory pathway containing a functional ATPS. Genes coding for putative standalone ATPS and APSK were indeed on the same locus in the genome of the strain DSM2095 that we had re-sequenced (Fig. 1d) (refs. ^13,23^). To confirm their functions, the ATPS and APSK from *M. thermolithotrophicus* (*Mt*ATPS and *Mt*APSK, respectively) were further characterized.

### A classic ATPS/APSK to activate ${\mathbf{SO}}_{\mathbf{4}}^{\mathbf{2-}}$

**Fig. 2:**
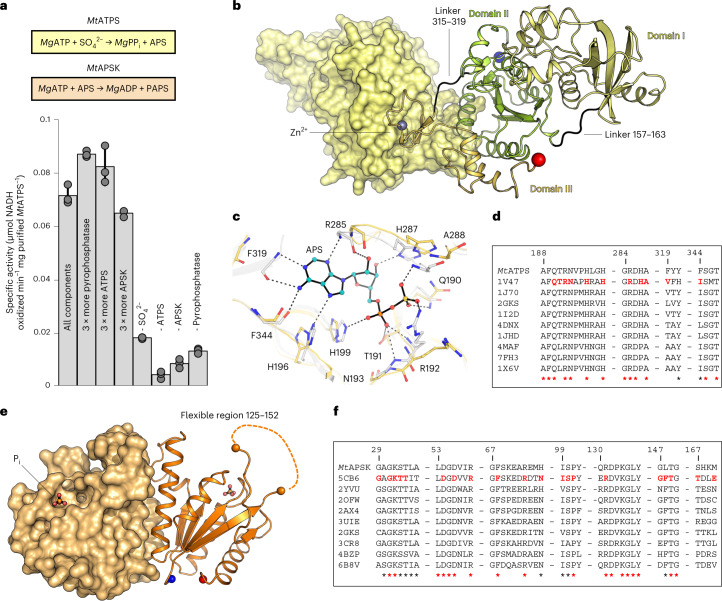
*Mt*ATPS and *Mt*APSK catalyse the first steps of the ${\mathbf{SO}}_{\mathbf{4}}^{{\mathbf{2-}}}$ reduction pathway. **a**, Top panel, reactions catalyzed by *Mt*ATPS and *Mt*APSK; Bottom panel, the specific activity of *Mt*ATPS and *Mt*APSK, determined via a coupled enzyme assay. ‘-’ indicates the absence of the indicated reactant. Data are presented as mean ± s.d. and individual values are shown as grey spheres (*n* = 3 replicates). **b**, *Mt*ATPS homodimeric structure in which one monomer is shown in light yellow surface and the other one in cartoon. **c**, Active site of *Mt*ATPS (yellow) superposed on the ATPS from *Thermus thermophilus* HB8 (PDB: 1V47, grey) containing the APS shown as balls and sticks with carbons coloured cyan. Residues involved in substrate binding are highlighted in sticks and only the ones from *Mt*ATPS are labelled. Hydrogen bonds between the ATPS from *T.* *thermophilus* and APS are represented as dashed lines. Nitrogen, oxygen, phosphorus and sulfur are coloured in blue, red, orange and yellow, respectively. **d**, Sequence conservation across ATPS homologues. **e,** *Mt*APSK homodimeric structure in which one monomer is shown in light orange surface and the other one in cartoon. The flexible loop illustrated by the dashed line could not be modelled. In all structures, the N and C termini are shown by a blue and red sphere, respectively. **f**, Sequence conservation across APSK homologues. For **d** and **f**, red bold residues are involved in substrate binding, while red and black stars are perfectly and well-conserved residues, respectively. Source data

The activity of the recombinantly expressed *Mt*ATPS and *Mt*APSK was tested via a coupled assay (Fig. 2a and Supplementary Fig. 1) and a specific activity of 0.070 ± 0.004 µmol of oxidized NADH min^−1^ mg^−1^ of *Mt*ATPS was measured. Under these conditions, the rate-limiting step was the pyrophosphatase activity. This highlights the need for rapid pyrophosphate degradation (Fig. 2a) to avoid a retro-inhibition as previously shown for other ATPS^24^. A ${{\rm{MoO}}}_{4}^{2-}$:${{\rm{SO}}}_{4}^{2-}$ ratio of 1:1.25 decreased the activity by half (see Methods), corroborating that ATPS is also reacting with ${{\rm{MoO}}}_{4}^{2-}$ as shown in other homologues^18,21^.

**Extended Data Fig. 3:**
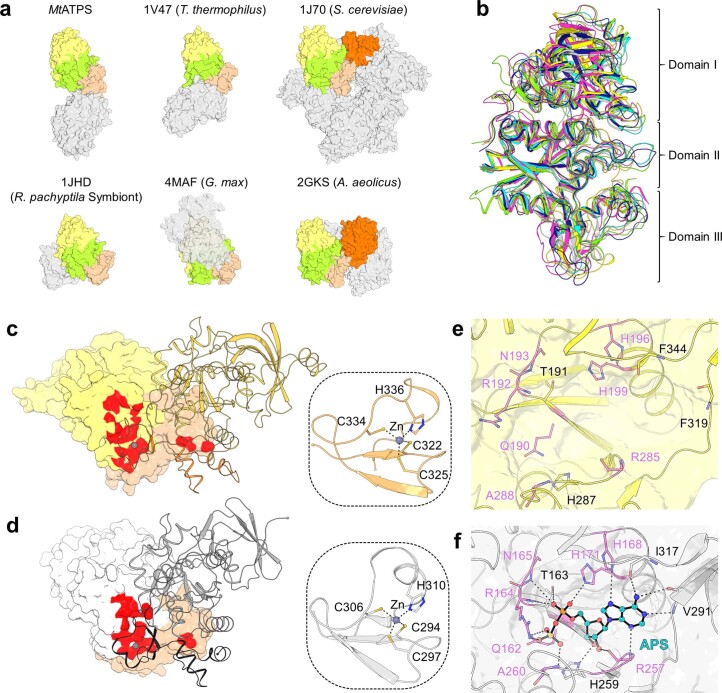
ATP-sulfurylase of *M. thermolithotrophicus* (*Mt*ATPS). **a**, Comparison of ATPS in surface representation, coloured by the domain composition I (yellow), II (light green), III (wheat) and APS kinase (dark orange). The grey surfaces corresponds to the opposing monomer. *Sc*ATPS organizes as homohexamer. **b**, Monomers of *Mt*ATPS (yellow), *Tt*ATPS (grey), *Sc*ATPS (navy blue), *Rrs*ATPS (magenta), *Gm*ATPS (green) and *Aa*ATPS (cyan) are superposed on domain II and shown as cartoons. Abbreviations and rmsd can be found in Supplementary Table 1. **c,d**, Surface area involved in the oligomerization of *Mt*ATPS (c) and *Tt*ATPS (d). One monomer is shown in surface representation and one monomer is displayed in cartoon. The monomer-monomer contacts, established by the domain III of one chain (in orange for *Mt*ATPS and black for *Tt*ATPS), are shown as a red surface. The wheat coloured surface highlights domain III. The framed inlet is a close up of the Zn binding motif and residues coordinating the Zn are drawn as sticks. Carbon, nitrogen and sulfur are coloured as orange/white, blue and yellow, respectively. **e**,**f**, Catalytic site of *Mt*ATPS (apo, e) and *Tt*ATPS with bound APS (f). Elements are coloured as in inlets (c, d) with oxygen and phosphorus in red and orange, respectively. Residues belonging to the canonical motifs of the ATPS are highlighted by pink coloured carbon atoms.

**Extended Data Fig. 4:**
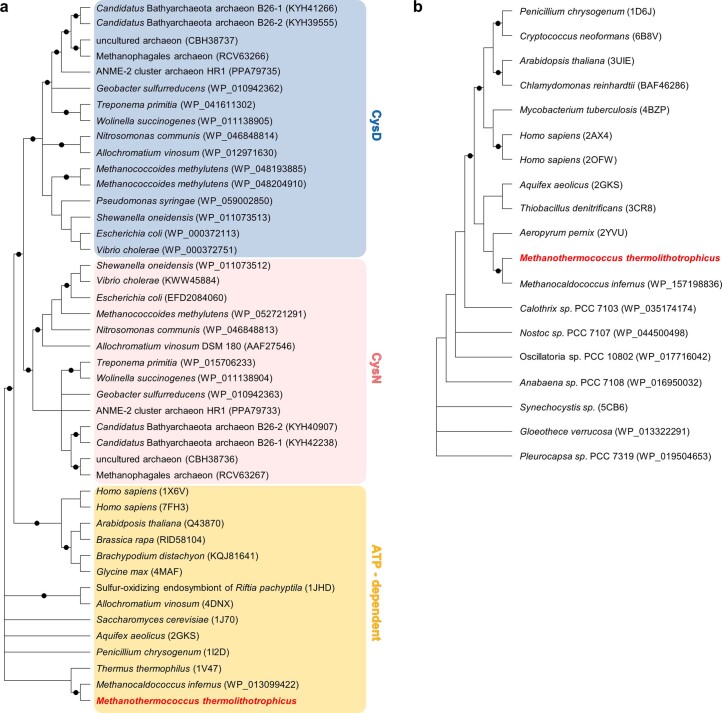
Phylogenetic analysis of *Mt*ATPS and *Mt*APSK. **a**, The heterodimeric sulfate adenylyltransferase (CysDN) with the homo-oligomeric ATP-dependent sulfurylase (sat) and **b**, APS-kinases. For panel a: the heterodimeric assimilatory ATP-sulfurylase is composed of a regulatory GTPase subunit CysN (light red) and a catalytic subunit CysD (blue). Sat is involved in both assimilatory and dissimilatory ${{\rm{SO}}}_{4}^{2-}$ reduction (light orange). *Mt*ATPS and *Mt*APSK are highlighted in bold red. Bootstrap support values ≥90 % are shown as dots on interior nodes.

The structure of *Mt*ATPS was refined to 1.97 Å resolution and obtained in an apo state despite co-crystallization with APS and ${{\rm{SO}}}_{4}^{2-}$ (Extended Data Table 1). While the crystal packing suggests a homotetrameric assembly in two crystalline forms, size exclusion chromatography and surface analysis using PISA (www.ebi.ac.uk/pdbe/pisa/) confirmed a homodimeric state similar to bacterial homologues (Extended Data Fig. 3a and Supplementary Fig. 2). The structure exhibits the typical ATPS fold comprising three domains (domain I, 1–156; domain II, 164–314 and domain III, 320–382; Fig. 2b). The dimeric interface is mainly organized by domain III as observed in *T. thermophilus*, a notable difference compared with other structural homologues (Extended Data Fig. 3a,b) (refs. ^25–27^). Similar to many thermophilic bacteria and archaea, the domain III contains a zinc-binding domain (320–343; Extended Data Fig. 3c,d) that might contribute to thermal stability^27^. *Mt*ATPS superposition with structural homologues shows a slight domain rearrangement probably due to the absence of substrate (Extended Data Fig. 3b). All residues critical for the reaction are conserved in *Mt*ATPS, arguing for a conserved reaction mechanism (Fig. 2c,d, Extended Data Figs. 3e,f and 4a, and Supplementary Fig. 3).

**Extended Data Fig. 5:**
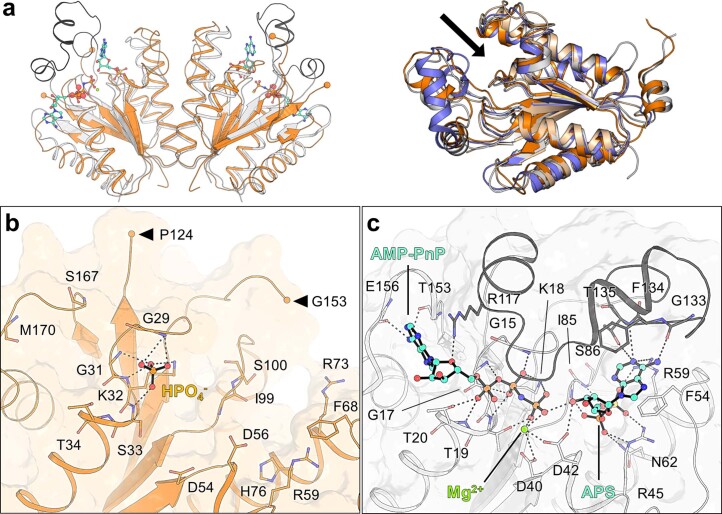
*Mt*APSK belongs to the APSK family. **a**, Left panel, homodimeric *Mt*APSK apo (orange) superposed to its closest homologue *Synechocystis* sp. PCC 6803 (*Ss*APSK, white, PDB: 5CB6) in complex with APS and AMP-PnP. The ligands are shown in sticks and spheres and the missing part 125–152 in *Mt*APSK (indicated by balls) is highlighted in black in *Ss*APSK. Carbon, nitrogen, oxygen, sulfur and phosphorus are coloured in light orange/white/cyan, blue, red, yellow and orange, respectively. Right panel: superposition of *Mt*APSK (wheat), *At*APSK (orange, PDB: 3UIE), *Ap*APSK (slate, PDB: 2YVU) and *Pc*APSK (white, PDB: 1M7H) on one monomer and shown in cartoon. The active site position is indicated by a black arrow. Abbreviations and rmsd can be found in Supplementary Table 2. **b, c**, Catalytic site of apo *Mt*APSK (b) and *Ss*APSK bound to APS and AMP-PnP (c). Elements are coloured as in (a), left panel. In (b), arrows indicate the missing part 125–152.

The APS-kinase model from *M*. *thermolithotrophicu*s, *Mt*APSK, was refined to 1.77 Å. *Mt*APSK forms a homodimer with an organization very similar to bacterial enzymes, which was expected due to its high sequence conservation (Extended Data Figs. 4b and 5a). Despite co-crystallization and soaking the crystals with APS and MgCl_2_, the *Mt*APSK structure was obtained in its apo state with a bound phosphate at the expected position of the ATP *β*-phosphate (Fig. 2e and Extended Data Fig. 5b,c). The N terminus and region 125–152 (the latter being involved in substrate binding^28,29^) could not be modelled due to the lack of electron density. However, the residues binding the substrates and Mg^2+^ are conserved (Fig. 2f, Extended Data Fig. 5b,c and Supplementary Fig. 4), suggesting that *Mt*APSK should be functional, as confirmed by the coupled enzyme assay.

### An exonuclease-derived PAP phosphatase

**Fig. 3:**
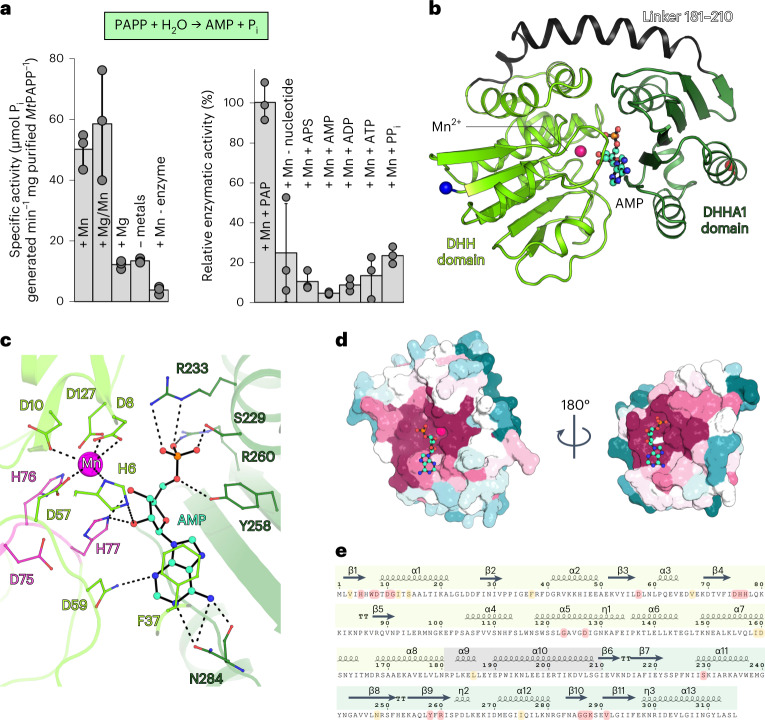
A unique type of PAP phosphatase. **a**, Top panel, reaction catalysed by *Mt*PAPP; Bottom panel, specific activity of the *Mt*PAPP determined via the production of P_i_ (left) and relative enzymatic activity towards different nucleotides (right). Data are presented as mean ± s.d. and individual values are shown as grey spheres (*n* = 3 replicates). **b**, Organization of *Mt*PAPP shown in cartoon representation. The N and C termini are highlighted as blue and red balls, respectively. Carbon, nitrogen, oxygen and phosphorus of AMP is coloured as cyan, blue, red and orange, respectively. **c**, Close-up view of the active site of *Mt*PAPP. The residues coordinating the AMP and Mn^2+^ ion are highlighted by sticks and coloured as in **b**, with the residues from the DHH motif coloured in pink. **d**, Cut-through view of *Mt*PAPP structure shown in surface representation and coloured by its sequence conservation across 168 archaeal homologues. The colour gradient ranges from variable (teal) to conserved (magenta). **e**, Secondary structure representation was done with ESPript 3.0 (ref. ^61^). The coloured frame corresponds to the different domains: DHH domain in light green, linker in grey and DHHA1 domain in darker green. Perfectly and well-conserved residues across 168 archaeal homologues are highlighted in red and yellow, respectively. The secondary structures composing *Mt*PAPP are labelled, in which β-sheets, α-helices, and β-turns are highlighted as arrows, springs and bold TT, respectively. Source data

If the ATPS and APSK are active, they will produce PAPS, an intermediate that could follow the metabolic routes 1b or 1c (Extended Data Fig. 1). Both routes will lead to the production of the toxic product PAP, which inhibits sulfotransferases and exoribonucleases, and disrupts RNA catabolism^30,31^. Therefore, it needs to be efficiently hydrolysed by a PAP phosphatase. While the genome did not contain any related PAP phosphatase, a gene coding for a putative phosphoesterase (Fig. 1d) was found in the genomic environment harbouring the ATPS and APSK genes. This PAP-phosphatase candidate, belonging to the DHH family, was recombinantly expressed and produced inorganic phosphate (P_i_) from PAP at fast rates (50.2 ± 5.9 µmol of P_i_ released min^−1^ mg^−1^ of purified enzyme with manganese). The activity was stimulated by manganese addition and showed a high specificity towards PAP (Fig. 3a).

**Extended Data Fig. 6:**
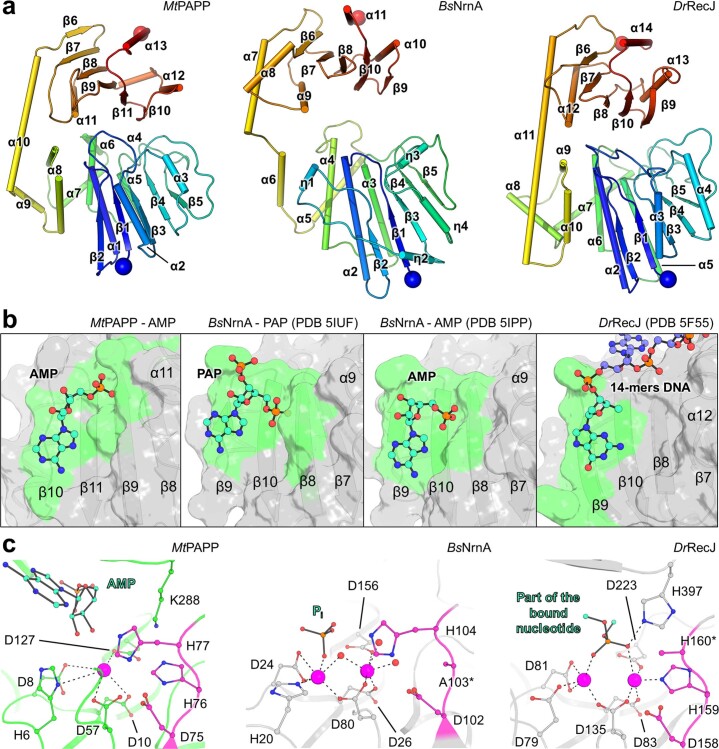
*Mt*PAPP shares similar structural features with exonucleases. **a**, Folding conservation across the *Mt*PAPP, the NanoRNase A from *Bacillus subtilis* (*Bs*NrnA, PDB: 5IUF) and the recombinase RecJ from *Deinococcus radiodurans* (*Dr*RecJ, PDB: 5F55). For *Bs*NrnA and *Dr*RecJ, the structures only represent the DHH and DHHA1 domains and the secondary structure motifs are renumbered to simplify the comparison with *Mt*PAPP. **b**, Differences in the nucleotide binding between *Mt*PAPP, NanoRNase A and the recombinase RecJ with the surface residues interacting with the ligand coloured in green. **c**, Close-up of the Mn^2+^ coordination between *Mt*PAPP, *Bs*NrnA (PDB: 5IZO) and *Dr*RecJ (PDB: 5F55). Mn^2+^ are shown as purple spheres and the residues coordinating them are highlighted as stick and balls. Carbon, nitrogen, oxygen and phosphorus atoms are coloured respectively in green/grey, blue, red and orange. Residues belonging to the canonical DHH-motif have pink coloured carbon atoms. The structure used for *Bs*NrnA is the His103Ala variant, and the side chain of the His160 has not been modelled in the *Dr*RecJ structure.

**Extended Data Fig. 7:**
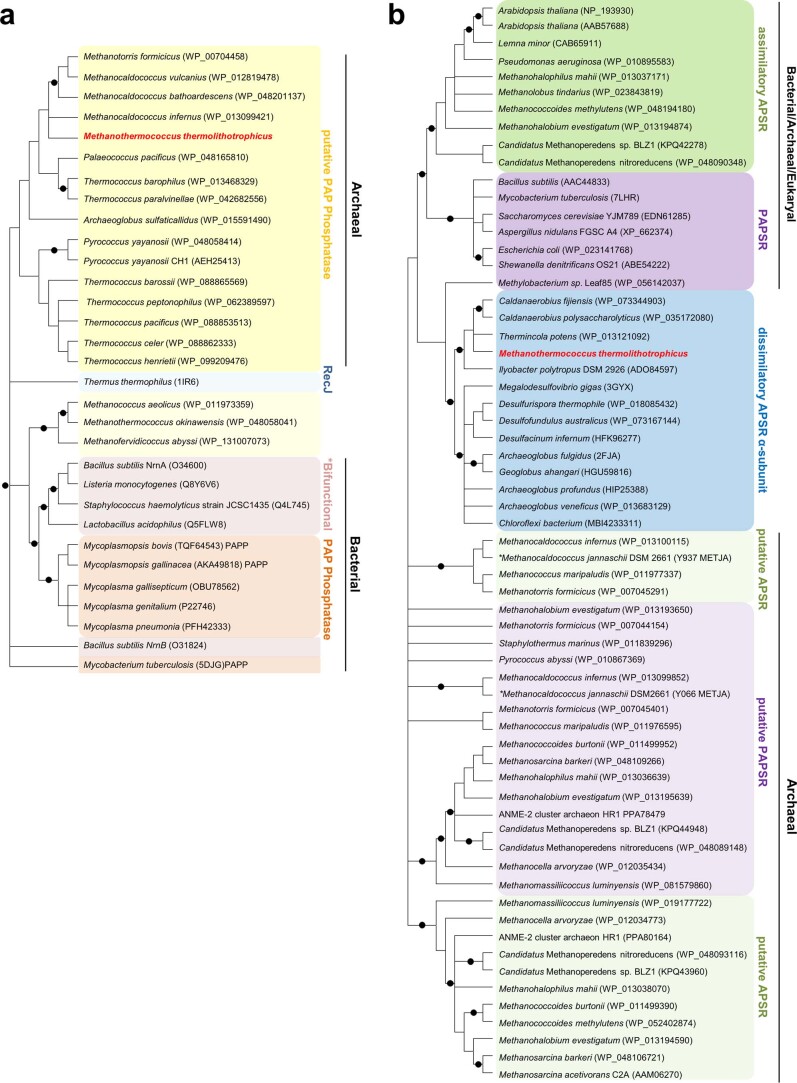
Phylogenetic analysis of *Mt*PAPP and *Mt*PAPSR. **a**, PAP-phosphatases with bifunctional oligoribonucleases and PAP-phosphatases, as well as exonucleases (RecJ), and **b**, dissimilatory APS-reductases (α-subunit) and (putative) assimilatory APS/PAPS-reductases. *Mt*PAPSR and *Mt*PAPP are highlighted in bold red. Bootstrap support values ≥90 % are shown as dots on interior nodes. Asteriks (*) in front of *Methanocaldococcus* *jannaschii* sequences highlight the two enzymes previously biochemically characterized.

To decipher the mechanism of this uncanonical PAP phosphatase (named *Mt*PAPP), the enzyme was co-crystallized with manganese and PAP. The structure, solved by molecular replacement with a template generated by AlphaFold2 (refs. ^32,33^), was refined to 3.1 Å resolution and contained the product AMP and an ion in its active site, modelled as a partially occupied Mn^2+^ (Extended Data Table 1). While the *Mt*PAPP sequence does not align with homologues belonging to the DHH family (except for the DHH motif), it shares an overall fold similar to the exonuclease RecJ or the oligoribonuclease NrnA from *Bacillus subtilis* (*Bs*NrnA, which also exhibits PAP-phosphatase activity; Extended Data Fig. 6a) (refs. ^34–36^). The monomer is composed of an N-terminal (DHH, residues 1–180) and a C-terminal domain (DHHA1, residues 211–315) interconnected by a linker region (residues 181–210), forming a central groove (Fig. 3b). The DHH domain contains the catalytic site and the DHHA1 domain serves as a scaffold to bind the substrate with high specificity (Fig. 3b,c and Extended Data Fig. 6b). The motif coordinating the Mn^2+^ ion in RecJ and *Bs*NrnA is perfectly conserved in *Mt*PAPP^34,36^, therefore we expect that in its active state, *Mt*PAPP would be loaded with two Mn^2+^. The first one, partially observed in the structure, is coordinated by four aspartates (Asp8, Asp10, Asp57, Asp127) and a long-range interaction with His6. The absent second Mn^2+^ would be coordinated by the Asp10, Asp57, Asp127, the DHH motif (His76, His77) as well as by water molecules (Extended Data Fig. 6c). While the AMP shares a similar localization with structural homologues (β9β10β11), it is bound by a different interaction with the protein (Extended Data Fig. 6b and Supplementary Fig. 5). The nucleotide binding site would ideally place the 3′-phosphate of the PAP in front of the manganese when the enzyme is in its closed state (Extended Data Fig. 6c). The inter-domain movement, allowed by the linker, would facilitate a rapid exchange of the substrate/product, increasing the turnover of *Mt*PAPP. The complete sequence of this PAP phosphatase was found in the genome of 168 archaea in which the nucleotide binding site is conserved (Fig. 3d,e and Supplementary Fig. 6). This suggests a common enzyme in archaea to detoxify PAP (Extended Data Fig. 7a).

### A dissimilatory APSR-like enzyme reduces PAPS

No genes encoding for a canonical PAPS reductase (route 1b) or sulfo-transferase (route 1c) were found in the *M. thermolithotrophicus* genome. However, genes annotated as dissimilatory APS reductase (α and β subunit, APSR; Extended Data Fig. 7b and Supplementary Fig. 7) are present and co-occur with the previously described genes (Fig. 1d).

**Fig. 4:**
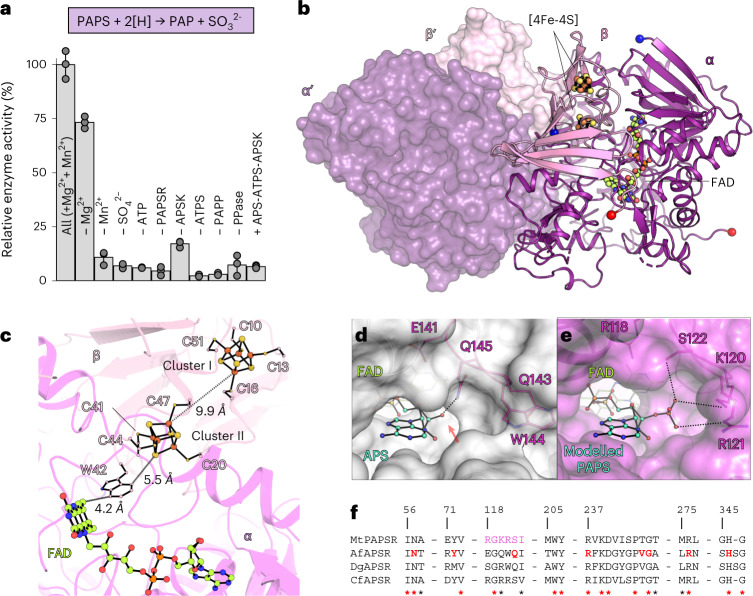
*Mt*PAPSR has a dissimilatory APS-reductase architecture but is specific to PAPS. **a**, Top panel, reaction catalyzed by *Mt*PAPSR; Bottom panel, relative enzyme activity of *Mt*PAPSR, determined via a coupled enzyme assay (Extended Data Fig. 8). Data are presented as mean ± s.d. and individual values are shown as grey spheres (*n* = 3 replicates). **b**, *Mt*PAPSR organization with one heterodimer in surface representation and the other in cartoon. N and C termini of both subunits are shown as balls and coloured in blue and red, respectively. Heterodimeric partners are labelled with a prime. Carbon, nitrogen, oxygen, phosphorus, iron and sulfur are coloured in lemon, blue, red, orange, brown and yellow, respectively. **c**, Close-up of cofactors and the electron flow. [4Fe-4S] clusters and cysteines coordinating them, FAD and the Trp42 proposed to participate in the electron transfer are shown in sticks and balls and coloured as in **b**. **d**,**e**, Active sites of APSR (**d**) from *A. fulgidus* containing APS (*Af*APSR, PDB: 2FJA) and *Mt*PAPSR with an artificially modelled PAPS (**e**) shown with a transparent surface. Residues involved in substrate recognition (based on modelled PAPS) are in balls and sticks and coloured as in **b**. A red arrow points to where PAPS would clash. **f**, Sequence conservation across the alpha subunit of *Mt*PAPSR, *Af*APSR, *Megalodesulfovibrio gigas* (*Dg*APSR, PDB: 3GYX) and the putative APSR from *Caldanaerobius fijiensis* (*Cf*APSR, WP_073344903), which shares 68% sequence identity with *Mt*PAPSR. Residues involved in APS binding for APSR are in bold and red; perfectly and well-conserved residues are highlighted with red and black stars, respectively. Trp206 and Tyr207 are involved in FAD binding. The sequence alignment was done with MUSCLE^62^. Source data

**Extended Data Fig. 8:**
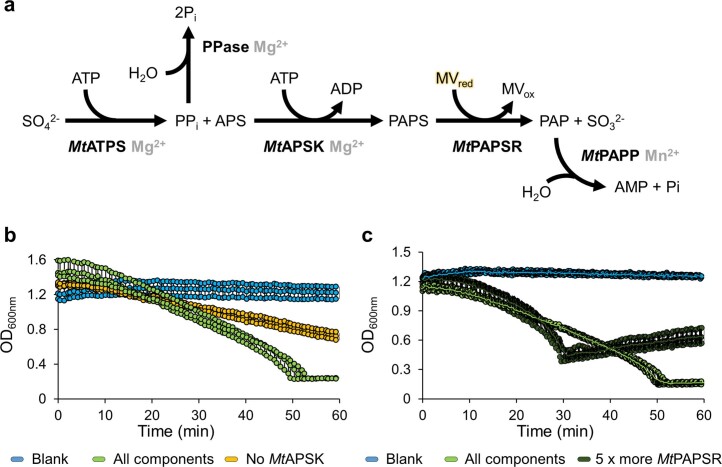
*In vitro* reconstitution of the ${\mathbf{SO}}_{\mathbf{4}}^{{\mathbf{2-}}}$ reduction pathway to measure *Mt*PAPSR activity. **a**, Scheme of the coupled enzyme assay used to measure *Mt*PAPSR activity via the oxidation of reduced methyl viologen (MV_red_) at 600 nm under an N_2_-atmosphere and at 45 °C. The enzymes were recombinantly expressed in *E*.*coli*, except for the pyrophosphatase, which was commercially obtained. The reaction mix contained all enzymes, metals (Mn^2+^, Mg^2+^), HEPES buffer at pH 7.0 and the substrates ${{\rm{SO}}}_{4}^{2-}$ and ATP. The reaction was started by the addition of ${{\rm{SO}}}_{4}^{2-}$. **b, c**, ‘Blank’ corresponds to a solution containing the buffer, the substrates, MV_red_ and dithionite but no enzymes. ‘All components’ contained every compound shown in scheme (a). **b**, Substrate specificity of *Mt*PAPSR. ‘No *Mt*APSK’ corresponds to all components except *Mt*APSK, which prevented the generation of PAPS from APS. With APS as the substrate, a specific enzymatic activity of 0.007 ± 0.001 µmol of oxidized MV.min^−1^.mg^−1^ of PAPSR was measured, which is only 6.54 % of the activity with the APSK (corresponds to Fig. 4a, +APS -ATPS -APSK). The difference can be attributed to an instability of the added APS. **c**, Impact of different concentrations of *Mt*PAPSR on the activity: A fivefold addition of *Mt*PAPSR resulted in a stimulation of the MV oxidation rate by 220 % (shown in dark green squares, ‘5 × more *Mt*PAPSR’). After 30 minutes the five-fold addition of the *Mt*PAPS−reductase led to aggregation, which is why the OD_600nm_ started to increase after this time point. All experiments were performed in triplicates and represented as data mean ± s.d. Source data

To experimentally confirm the activity and substrate specificity of this APS-reductase-like enzyme, both subunits were co-expressed in *Escherichia coli*, purified and tested for enzyme activity assays (Fig. 4a). In contrast to dissimilatory APSRs which catalyse the reversible reduction of APS to AMP and ${{\rm{SO}}}_{3}^{2-}$ (refs. ^37,38^), we could not measure the reverse reaction (that is, AMP and ${{\rm{SO}}}_{3}^{2-}$, or PAP and ${{\rm{SO}}}_{3}^{2-}$ as substrates) for *M. thermolithotrophicus* enzyme by using K_3_Fe(CN)_6_ as an electron acceptor. Instead, we used a coupled enzyme assay to reconstitute the pathway in vitro (Extended Data Fig. 8). *Mt*ATPS, a pyrophosphatase and the *Mt*APSK were used to generate PAPS and *Mt*PAPP was added to remove PAP, a potential retro-inhibitor of the reaction^39^. The activity was monitored via the oxidation of reduced methyl viologen (MV_red_). When all components were present, a specific enzymatic activity of 0.114 ± 0.007 µmol of oxidized MV min^−1^ mg^−1^ of the APS-reductase-like enzyme was measured. A fivefold excess of the APS-reductase-like enzyme resulted in a 220% increase of the specific enzyme activity, indicating that the enzyme was the rate-limiting step of the reaction (Extended Data Fig. 8c). However, the accumulation of PAP (induced by the removal of *Mt*PAPP or Mn^2+^) strongly inhibited the activity. The specific enzymatic activity with APS as a substrate (that is, removal of *Mt*APSK) was 0.007 ± 0.001 µmol of oxidized MV min^−1^ mg^−1^ of the APS-reductase-like enzyme (Fig. 4a). Considering the complexity of this coupled enzyme assay, kinetic parameters could not be determined. However, the assay did provide insights about the substrate specificity and confirmed that the APS-reductase-like enzyme from *M. thermolithotrophicus* exhibits traits of a PAPS reductase.

**Extended Data Fig. 9:**
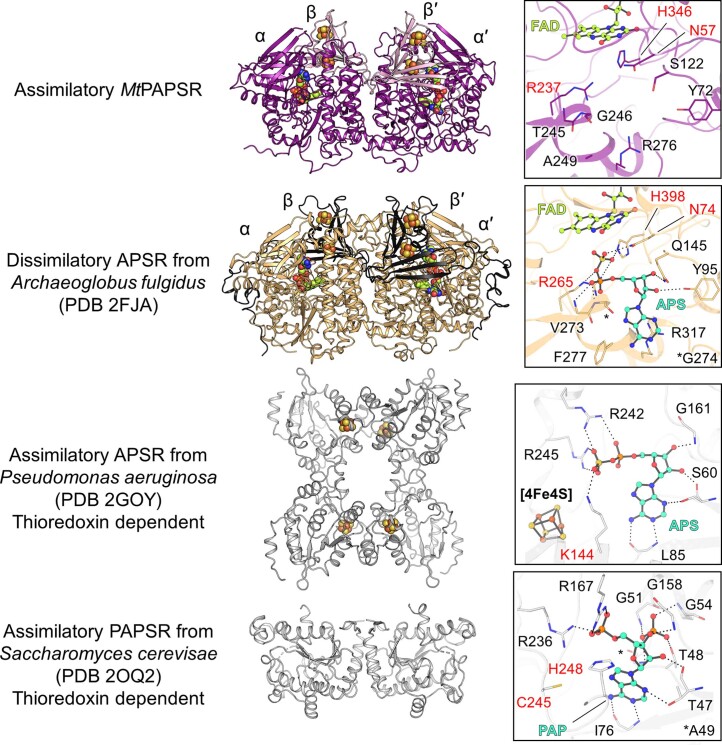
Comparison of a dissimilatory APS-reductase and assimilatory P/APS-reductases with the *Mt*PAPSR. All structures are shown in cartoon with their (metallo)-cofactors in balls and sticks. Close-ups of the active sites (on the right) with residues important for substrate binding are highlighted as balls and sticks. The dissimilatory APS-reductase from *Archaeoglobus fulgidus* is a heterotetramer, composed of two (αβ)-subunits. Each β-subunit contains two [4Fe-4S]-cluster and each α-subunit one FAD. The presented assimilatory APSR from *Pseudomonas aeruginosa* is a homotetramer. It contains one [4Fe-4S]-cluster per monomer. The assimilatory PAPSR from *Saccharomyces cerevisiae* is homodimeric. The elements oxygen, nitrogen, phosphorus, sulfur and iron are coloured red, blue, orange, yellow and brown, respectively. The carbon of the substrate/product is coloured in cyan, and in yellow for the FAD. Catalytic residues are highlighted with red labels.

To gain further molecular insights into the unconventional APS-reductase-like enzyme from *M. thermolithotrophicus*, the enzyme was crystallized under anaerobic conditions. The structure was solved by a single-wavelength anomalous dispersion experiment measured at the Fe K-edge and refined to 1.45 Å resolution (Extended Data Table 1). The complex organizes as an α2β2 heterotetramer, with the same assembly as dissimilatory APS reductases (Fig. 4b and Extended Data Fig. 9). It is, however, drastically different from characterized single-domain assimilatory APS/PAPS reductases, which are thioredoxin/glutathione-dependent. Assimilatory APS/PAPS reductases share no sequence or structural homology with the *M. thermolithotrophicus* enzyme, and several motifs that are proposed to mediate substrate binding and catalytic activity in assimilatory APS/PAPS reductases are absent (Extended Data Fig. 9) (ref. ^40^).

In *M. thermolithotrophicus*, the α subunit, containing the flavin adenine dinucleotide (FAD), is a member of the fumarate reductase family^37,41,42^ and the β subunit is mainly composed of a ferredoxin-like domain in which two [4Fe-4S] clusters are coordinated by eight cysteine residues (Fig. 4c). While there are no assimilatory P/APS-reductase homologues to *M. thermolithotrophicus* enzyme, it shares 38% sequence identity with the α subunit of the dissimilatory APS reductase from *A. fulgidus* (*Af*APSR PDB: 2FJA, rmsd of 1.02 Å for 437 C*α* aligned on the α subunit). The residues coordinating APS, invariable in the dissimilatory family, differ in *M. thermolithotrophicus* and might provoke a switch of specificity from APS to PAPS. Despite a short soak with PAP, the putative substrate pocket contains only solvent, and we used the *Af*APSR to artificially model PAPS in the active site of the enzyme (Fig. 4d,e). The different substitutions mainly carried by loop 104–123 would accommodate the additional 3′-phosphate group by salt-bridge interactions and hydrogen bonds (Fig. 4d–f). In APS reductases, however, a conserved glutamine (α145 in *A. fulgidus*) would clash with this phosphate group. The catalytic residues proposed in dissimilatory APS reductases are retained in the enzyme of *M. thermolithotrophicus* (Extended Data Fig. 9 and Supplementary Fig. 7). We therefore propose an identical reaction mechanism on the basis of a nucleophilic attack of the atom N5 of FAD on the sulfur PAPS, which creates a FAD-PAPS intermediate that decays to PAP and FAD-${{\rm{SO}}}_{3}^{2-}$ (refs. ^37,42^). Taking together the enzyme rates and the structural analysis, we propose that *M. thermolithotrophicus* harbours a unique class of PAPS reductase (*Mt*PAPSR) used to convert PAPS into ${{\rm{SO}}}_{3}^{2-}$ and PAP.

### F_420_-dependent sulfite reductase catalyses the last step of the pathway

The ${{\rm{SO}}}_{3}^{2-}$ generated by *Mt*PAPSR must be further reduced to HS^−^. In hydrogenotrophic methanogens, ${{\rm{SO}}}_{3}^{2-}$ damages the methane-generating machinery and must be detoxified by the F_420_-dependent sulfite reductase (Fsr)^23,43^. We previously identified and characterized Group I Fsr in *M. thermolithotrophicus* (*Mt*Fsr) and determined a robust enzymatic activity towards ${{\rm{SO}}}_{3}^{2-}$ (ref. ^23^). Besides a second Fsr isoform, *M. thermolithotrophicus* does not contain other potential sulfite reductases. While mass spectrometry confirmed that the Fsr isolated from ${{\rm{SO}}}_{4}^{2-}$-grown cells is the characterized Group I *Mt*Fsr, the physiological role of the second Fsr isoform remains unknown^23^. Therefore, *Mt*Fsr is the best candidate to catalyse the final reduction of ${{\rm{SO}}}_{3}^{2-}$ to HS^−^. Native polyacrylamide gel electrophoresis (native PAGE) with cell extracts of cultures grown on different sulfur substrates confirmed the absence of *Mt*Fsr from cells grown on Na_2_S and its high abundance in cells grown on ${{\rm{SO}}}_{3}^{2-}$ (refs. ^23,43^).

**Fig. 5:**
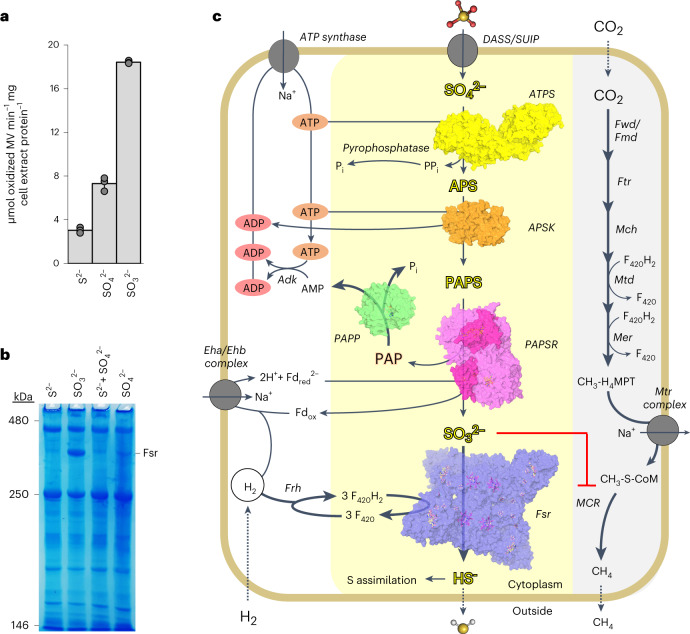
Proposed ${\mathbf{SO}}_{\mathbf{4}}^{{\mathbf{2-}}}$ assimilation pathway in a methanogen. **a**, Sulfite-reductase activity in cell extract from *M. thermolithotrophicus* grown on different sulfur sources. Data are presented as mean ± s.d. and individual values are shown as grey spheres (*n* = 3 biologically independent replicates). **b**, hrCN gel with *M. thermolithotrophicus* cell extract grown on different sulfur sources (15 µg protein loaded per sample, *n* = 2 biologically independent duplicates). **c**, Proposed ${{\rm{SO}}}_{4}^{2-}$ assimilation pathway in *M. thermolithotrophicus*. Yellow and grey backgrounds highlight the ${{\rm{SO}}}_{4}^{2-}$ reduction and methanogenesis pathways, respectively. Thick arrows indicate high metabolic fluxes. The structures of the enzymes operating the ${{\rm{SO}}}_{4}^{2-}$-assimilation pathway are shown in surface representation, with ligands as balls and sticks. Enzymes are abbreviated as follows: Fwd/Fmd, formylmethanofuran dehydrogenases; Ftr, tetrahydromethanopterin (H_4_MPT) formyltransferase; Mch, methenyl-H_4_MPT cyclohydrolase; Mtd, methylene-H_4_MPT dehydrogenase; Mer, 5,10-methylene-H_4_MPT reductase; Mtr, N^5^-CH_3_-H_4_MPT: coenzyme M methyl-transferase; Mcr, methyl-coenzyme M reductase; Adk, adenylate kinase; Frh, F_420_-reducing [NiFe]-hydrogenase; Eha/Ehb, energy-converting hydrogenase. The putative ${{\rm{SO}}}_{4}^{2-}$ transporters belonging to the class DASS/SUIP are proposed to be WP_018154444/WP_018154062 and the pyrophosphatase WP_018154121. Source data

We determined a specific sulfite reductase activity of 18.42 ± 0.13 µmol of oxidized MV min^−1^ mg^−1^ of cell extract from Na_2_SO_3_-grown cells, in comparison to 7.31 ± 0.63 µmol of oxidized MV min^−1^ mg^−1^ of cell extract from Na_2_SO_4_-grown cells, whereas cell extract from an Na_2_S-grown culture had a specific sulfite reductase activity of 3.04 ± 0.25 µmol of oxidized MV min^−1^ mg^−1^ (Fig. 5a). In agreement, we observed a band compatible with Fsr on the native PAGE for the ${{\rm{SO}}}_{4}^{2-}$-grown culture but in lower amounts compared with ${{\rm{SO}}}_{3}^{2-}$ conditions (Fig. 5b). The *Mt*Fsr structure recently published by our group was obtained from ${{\rm{SO}}}_{4}^{2-}$-grown cells, which confirmed that it is the same enzyme expressed as under ${{\rm{SO}}}_{3}^{2-}$ conditions^23^. Taken together, these results argue that *Mt*Fsr is used as the last enzyme in the ${{\rm{SO}}}_{4}^{2-}$ reduction pathway (Fig. 5c).

### Genetic potential is not enough to sustain ${\mathbf{SO}}_{\mathbf{4}}^{\mathbf{2-}}$ growth

**Fig. 6:**
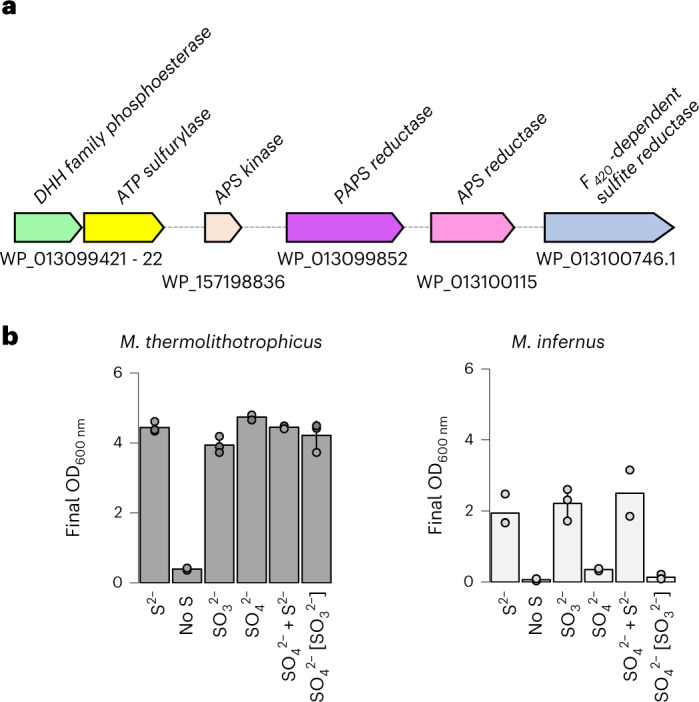
${\mathbf{SO}}_{\mathbf{4}}^{{\mathbf{2-}}}$ reduction potential in *Methanococcales*. **a**, *Methanocaldococcus infernus* has the genomic potential to perform the whole ${{\rm{SO}}}_{4}^{2-}$ assimilation pathway. WP_013099421 has 59.68% amino acid sequence identity with the *Mt*PAPP, WP_013099422 has 70.60% sequence identity with *Mt*ATPS and WP_157198836 has 75.44% sequence identity with *Mt*APSK. The APSR (WP_013100115) and PAPSR (WP_013099852) are similar to the biochemically characterized APSR and PAPSR from *M. jannaschii* (68.64% and 58.35% amino acid sequence identity, respectively), which have been shown to reduce APS/PAPS^44,45^. WP_013099852 is not homologous to *Mt*PAPSR but homologous to WP_018154242, a putative PAPS reductase in *M. thermolithotrophicus*. WP_013100746 has 65.30% sequence identity to Group I *Mt*Fsr. **b**, Growth of *M. thermolithotrophicus* and *M. infernus* on 2 mM Na_2_S, without an additional sulfur source, 2 mM Na_2_SO_3_, 2 mM Na_2_SO_4_ or 2 mM Na_2_SO_4_ with 2 mM Na_2_S. The ${{\rm{SO}}}_{3}^{2-}$ in brackets indicates that it was used as the sulfur substrate for the inoculum. Represented are the maximum OD_600nm_ of the cultures, in triplicates, shown as mean ± s.d. *M. infernus* cultures grown without sulfur,Na_2_SO_4_ and on Na_2_S with Na_2_SO_4_ are in duplicates. The individual data point of each replicate is shown as a sphere. Source data

Methanogens commonly use HS^−^ as a sulfur source, and the ones that express Fsr type I can also grow on ${{\rm{SO}}}_{3}^{2-}$ (refs. ^13,23,43^). Interestingly, some methanogens have genes that encode for proteins of the complete or partial ${{\rm{SO}}}_{4}^{2-}$ reduction pathway (Supplementary Fig. 8) (ref. ^13^). So why is *M. thermolithotrophicus* the only methanogen so far that has been proven to grow on ${{\rm{SO}}}_{4}^{2-}$? We used *Methanocaldococcus infernus* as a model organism to investigate this further. *M. infernus* is a marine hyperthermophile that shares a very similar physiology with *M. thermolithotrophicus* and can grow in the same medium. It contains all genes coding for the enzymes characterized in this study except for the described PAPSR. However, the *M. infernus* genome encodes for a putative thioredoxin-dependent PAPSR and APSR, which share high sequence identities with the biochemically characterized assimilatory APSR and PAPSR from *M. jannaschii* (Fig. 6a and Extended Data Fig. 7b) (refs. ^44,45^). Therefore, based on genomic information, *M. infernus* should be able to assimilate ${{\rm{SO}}}_{4}^{2-}$.

*M. thermolithotrophicus* and *M. infernus* were grown in the same medium and under the same cultivation conditions except that *M. infernus* was kept at 75 °C and *M. thermolithotrophicus* at 65 °C. *M. infernus* grew on 2 mM Na_2_S and Na_2_SO_3_ but was unable to use ${{\rm{SO}}}_{4}^{2-}$ as a sole source of sulfur in contrast to *M. thermolithotrophicus* (Fig. 6b).

This raises the question about the physiological function of the genes related to ${{\rm{SO}}}_{4}^{2-}$ assimilation in methanogenic archaea. Based on our data, it could be that other methanogens still require these enzymes to acquire sulfur via the route 1c (Extended Data Fig. 1). The sulfur group would be transferred to an acceptor by a non-canonical sulfo-transferase, which might be important for uncharted biosynthetic pathway(s). This could explain why the gene coding for the PAPP is still present in methanogens also harbouring the genes encoding an ATPS as well as an APSK. A counterargument to this hypothesis is the presence of the thioredoxin-dependent PAPSR or APSR, characterized in *M. jannaschii*, which rather argues for route 1b (refs. ^13,44,45^). It is worth noting that the gene coding for this putative assimilatory APSR also exists in *M. thermolithotrophicus* (WP_018154242.1). Therefore, further biochemical investigations will be needed to elucidate the physiological roles of these enzymes in methanogens.

## Discussion

This work unveiled the unique ${{\rm{SO}}}_{4}^{2-}$-assimilation metabolism of a methanogenic archaeon, offering a molecular snapshot of the complete set of enzymes involved in the pathway. *M. thermolithotrophicus* activates ${{\rm{SO}}}_{4}^{2-}$ by conventional ATPS and APSK, but transforms it further by uncanonical enzymes (Fig. 5c).

PAPS produced by the APSK is usually metabolized by thioredoxin- or glutathione-dependent assimilatory PAPS reductases, which are organized as homo-oligomers. In contrast, *Mt*PAPSR inherited the heterotetrameric organization and FAD-based catalytic mechanism from dissimilatory APS reductases (Fig. 4, and Extended Data Figs. 1 and 9). We propose that the substitution of only a few amino acids switched the specificity towards PAPS (Fig. 4d–f), which might have been the result of a fine-tuned evolutionary adaptation to promote assimilatory ${{\rm{SO}}}_{4}^{2-}$ reduction. It would be worthwhile to exchange the residues that confer PAPSR traits at the active site (Ser122, Lys120, Arg121) with those of APSR and observe the effects on substrate affinity.

The generated PAP is efficiently hydrolysed by *Mt*PAPP. This PAP phosphatase belongs to the DHH family of phosphoesterases and shares structural homology with exonucleases but has no sequence homology with them. In comparison, conventional PAP phosphatases (part of the FIG superfamily) have a different fold (that is, CysQ) and use three magnesium ions to hydrolyse the 3′-phosphate of PAP^30^. *Mt*PAPP appears to be a remarkable example of convergent evolution, illustrating how archaea developed their own apparatus to detoxify PAP efficiently.

Group I Fsr catalyses the final step of the ${{\rm{SO}}}_{4}^{2-}$ reduction pathway. This enzyme shows distinct traits of dissimilatory sulfite reductases, with the active site composition of an assimilatory one^23^. By encoding the *fsr* gene on a different locus and most probably under a different regulator for its expression (for example, sulfite sensor), the methanogen is able to uncouple rapid ${{\rm{SO}}}_{3}^{2-}$ detoxification from expressing the whole ${{\rm{SO}}}_{4}^{2-}$ assimilation machinery. While the *Mt*ATPS, *Mt*APSK and *Mt*PAPSR show rather slow catalytic rates (see Supplementary Discussion), *Mt*Fsr and the *Mt*PAPP have high specific activities compared with the first steps, triggering the equilibrium towards HS^−^ production and efficiently eliminating toxic intermediates. Although our proposed pathway (Fig. 5c) would allow favourable thermodynamics, the first reactions should be regulated to avoid unnecessary ATP hydrolysis. We suspect that *Mt*ATPS, *Mt*APSK and *Mt*PAPSR are cross-regulated by the accumulation of their own products, as already shown for homologues^39,46,47^, which would allow direct retro-control to harmonize the intracellular sulfur flux.

*M. thermolithotrophicus* lives at the thermodynamic limit of life but the described ${{\rm{SO}}}_{4}^{2-}$ assimilation requires the hydrolysis of three ATP to ADP for one processed ${{\rm{SO}}}_{4}^{2-}$. Nevertheless, it is expected that under natural conditions, the benefit of fixing ${{\rm{SO}}}_{4}^{2-}$ counterbalances the energy expenditure. The ${{\rm{SO}}}_{4}^{2-}$-grown cultures are not hampered by the additional energy requirement, which can be explained by our cultivation conditions that provide a high and constant H_2_ partial pressure. Under environmental conditions, with a lower and fluctuating H_2_ partial pressure, growth on ${{\rm{SO}}}_{4}^{2-}$ is likely to be more challenging for *M. thermolithotrophicus*. While the methanogen cannot avoid the ATP investment, it may have found an energy-saving strategy for the 8-electron reduction reaction from PAPS to HS^−^. Fsr oxidizes F_420_H_2_, which is reduced back by the F_420_-reducing hydrogenase^23,48^. F_420_H_2_ or NAD(P)H would be advantageous electron donors for *Mt*PAPSR, but it would require the assistance of an oxidase partner that has not yet been identified. Alternatively, the standalone *Mt*PAPSR may depend on reduced ferredoxin, which could be obtained from the H_2_-dependent ferredoxin reduction via the Eha/Ehb complex, another advantageous strategy of hydrogenotrophs to provide reducing power to fuel anabolic reactions (proposed in Fig. 5c) (ref. ^49^).

So far, it appears that the concomitant process of methanogenesis and complete ${{\rm{SO}}}_{4}^{2-}$ reduction to HS^−^ is restricted to *M. thermolithotrophicus*. Strikingly, the only apparent difference between *M. thermolithotrophicus* and other methanogens with the genomic potential to perform ${{\rm{SO}}}_{4}^{2-}$ reduction is the acquisition of a PAPS reductase, which appears to belong to the dissimilatory family (Supplementary Fig. 8, Extended Data Fig. 7b and Supplementary Discussion). The physiological function of these ${{\rm{SO}}}_{4}^{2-}$-reduction-associated genes in other methanogens remains to be uncovered, as well as the advantages of assimilating ${{\rm{SO}}}_{4}^{2-}$ for *M. thermolithotrophicus*. From an ecological point of view, it might be beneficial, if not essential, for *M. thermolithotrophicus* survival to be able to switch from H_2_S uptake to ${{\rm{SO}}}_{4}^{2-}$ reduction under environmental conditions (see Supplementary Discussion).

The transplantation of the *M. thermolithotrophicus* ${{\rm{SO}}}_{4}^{2-}$ reduction system into methanogenic hosts, which are already used as gas converters (for example, *Methanothermobacter*), would circumvent the need for highly toxic and explosive H_2_S by using inexpensive and abundant ${{\rm{SO}}}_{4}^{2-}$. Beyond opening fantastic possibilities for safer biotechnological applications, a ${{\rm{SO}}}_{4}^{2-}$-reducing hydrogenotrophic methanogen also reinforces the question about the extent of an intertwined methanogenesis and sulfate reduction pathway during the evolution of early archaea. *M. thermolithotrophicus* has most probably assembled the entire ${{\rm{SO}}}_{4}^{2-}$ reduction pathway progressively via a ‘mix-and-match’ scenario, providing a competitive advantage under fluctuating sulfur-source conditions and expanding its ecological niches.

## Methods

### Archaea strains and cultivation media

*M. thermolithotrophicus* (DSM 2095), *M. infernus* (DSM 11812) and *A. fulgidus* (DSM 4304) cells were obtained from the Leibniz Institute DSMZ-German Collection of Microorganisms and Cell Cultures (Braunschweig, Germany). *M. thermolithotrophicus* and *M. infernus* were cultivated in the same previously described minimal medium with some modifications^23^ (see Extended Data for the complete composition of the media).

### Anaerobic growth of Archaea

Cell growth was followed spectrophotometrically by measuring the OD_600_. The purity of the culture was checked by light microscopy. The methanogens were cultivated with 1 × 10^5^ Pa of H_2_:CO_2_ at an 80:20 ratio in the gas phase. *M. infernus* was cultivated at 75 °C in 250 ml glass serum flasks and *M. thermolithotrophicus* was grown at 65 °C in flasks or fermenters. The serum flasks were not shaken but standing. *A. fulgidus* was cultivated in anaerobic and sealed 22 ml Hungate tubes, with 0.8 × 10^5^ Pa N_2_:CO_2_. DSM 4304 culture (0.5 ml) was grown in 10 ml of classic media (see Supplementary Materials for the complete composition of the media composition) containing a final concentration of 20 mM d/l-lactate. The culture was incubated at 80 °C, standing. All cultures were stored at room temperature in the dark under anaerobic conditions. For the *A. fulgidus* medium, we found that high molybdate concentrations made it unstable. One of the bottles with a high MoO_4_^2−^ concentration turned yellow (unrelated to O_2_ contamination) and was omitted, resulting in triplicate instead of quadruplicate cultures (Fig. 1c, right panel).

### Adaptation of *M. thermolithotrophicus* to ${\mathbf{SO}}_{\mathbf{4}}^{\mathbf{2-}}$ and minimal ${\mathbf{SO}}_{\mathbf{4}}^{\mathbf{2-}}$ requirement

*M. thermolithotrophicus* cells grown on 2 mM Na_2_S were successively transferred to 10 ml sulfur-free cultivation medium. After two transfers, the carry-over sulfur concentration of the inoculum did not support growth of *M. thermolithotrophicus*. By supplementing 2 mM Na_2_SO_4_, *M. thermolithotrophicus* growth was resumed. No reducing agent was added to cope with the absence of HS^−^, which normally establishes a suitable reducing environment. Incubation without shaking is particularly important for reproducibility. Therefore, after inoculation, the cultures were incubated at 65 °C, standing for one night followed by shaking at 180 revolutions per minute (r.p.m.) until they reached their maximum OD_600_. The gas phase was refreshed after the overnight incubation to maintain the pressure at 1 × 10^5^ Pa of H_2_:CO_2._ To measure the minimal ${{\rm{SO}}}_{4}^{2-}$ concentration required to sustain growth, sulfur-limited *M. thermolithotrophicus* cells (using an inoculum to medium ratio of 1:20) were provided with 2 mM, 1 mM, 0.5 mM, 0.25 mM, 0.1 mM and 0.04 mM Na_2_SO_4_. Growth was still observable for cells grown on 0.1 mM but not on 0.04 mM Na_2_SO_4_.

### ${\mathbf{SO}}_{\mathbf{4}}^{\mathbf{2-}}$ measurements via ion chromatography

Ion chromatography (Methrom ion chromatograph) was used to measure the ${{\rm{SO}}}_{4}^{2-}$ concentrations, analysed via the software IC MagIC Net 3.2. A volume of 8 ml per sample was required, with a maximum concentration of 0.5 mM ${{\rm{SO}}}_{4}^{2-}$. ${{\rm{SO}}}_{4}^{2-}$-reducing *M. thermolithotrophicus* cells were therefore grown in 1 l Duran bottles with 100 ml sulfur-free media, which was supplemented with 0.5 mM ${{\rm{SO}}}_{4}^{2-}$ before inoculation. As a negative control, 0.5 mM Na_2_S-grown *M. thermolithotrophicus* cells were used, inoculated and collected similarly as the ${{\rm{SO}}}_{4}^{2-}$-reducing cultures. All samples were taken aerobically and were passed through a 0.45 µM filter (Sartorius). If the cell densities were too high to be filtered, the samples were centrifuged at 13,000 × *g* for 7 min at 4 °C and the supernatant was taken for ion chromatography measurements. The samples were stored at 4 °C if the measurements were not immediately performed.

### Growth of *M. thermolithotrophicus* in a fermenter

*M. thermolithotrophicus* was grown in three independent fermenters at 60 °C, with 10 mM Na_2_SO_4_ as sole sulfur source. For each fermenter, 7 l of anaerobic cultivation medium (see Sulfur-free cultivation medium for *Methanococcales*) supplemented with 10 mM Na_2_SO_4_ was continuously bubbled with H_2_:CO_2_ (80:20, 3 l min^−1^). Under stirring (220 r.p.m.), the medium was inoculated with 360 ml preculture (with an OD_600_ higher than 3). One hour after inoculation, the culture was stirred at 800 r.p.m. NaOH (1 M) was used as a base to readjust the pH upon acidification, which was controlled using a pH probe. The cells were grown until late exponential phase (OD_600_ of 6.25–6.8) and then immediately transferred in an anaerobic tent (N_2_:CO_2_ atmosphere at a ratio of 90:10). Cells were collected by anaerobic centrifugation for 30 min at 6,000 × *g* at 4 °C. The highest OD_600nm_ recorded for *M*. *thermolithotrophicus* in a SO_4_^2−^-grown fermenter was 6.8 after 20 h. ${{\rm{SO}}}_{4}^{2-}$ Culture (7 l) with an OD_600_ of 6.8 yielded 54 g of cells (wet weight). The cell pellet was transferred in a sealed bottle, gassed with 0.3 × 10^5^ Pa N_2_, flash frozen in liquid N_2_ and stored at −80 °C.

### Synthetic gene constructs

The DNA sequences of the ATP sulfurylase, the APS kinase, the PAP phosphatase and the PAPS reductase α and β subunits from *M. thermolithotrophicus* were codon optimized for *E. coli*, synthesized and cloned into pET-28a(+) vectors. For *Mt*ATPS, *Mt*APSK and *Mt*PAPP, the restriction sites NdeI and BamHI were used, with a stop codon (TGA) incorporated before BamHI. For *Mt*PAPSR, a His-tag was placed at the C terminus of the α subunit and a ribosome binding site was inserted between the coding sequences of the α and β subunits. The *Mt*PAPSR construct had the restriction sites NcoI and BamHI, with one stop codon incorporated after the His-tag for the α subunit and one stop codon before BamHI for the β subunit. These steps were performed by GenScript (GenScript). All sequences used are detailed in Supplementary Information under Constructs and gene codon optimization.

### Enzyme overexpression and purification

All constructs were overexpressed and purified under aerobic conditions following a similar protocol, except for *Mt*PAPSR which was overexpressed and purified under an anaerobic atmosphere. All enzymes were passed on a HisTrap high-performance column (GE Healthcare), followed, if necessary, by tag cleavage and gel filtration (see Supplementary Materials for the complete protocol).

### Protein crystallization

Purified *Mt*ATPS, *Mt*APSK and *Mt*PAPP were kept in 25 mM Tris/HCl pH 7.6, 10% v/v glycerol, 2 mM dithiothreitol and 150 mM NaCl. *Mt*PAPSR was kept in the same buffer without NaCl. Freshly prepared unfrozen samples were immediately used for crystallization. *Mt*ATPS, *Mt*APSK and *Mt*PAPP crystals were obtained under aerobic conditions at 18 °C. *Mt*PAPSR crystals were obtained anaerobically (N_2_:H_2_, gas ratio of 97:3) by initial screening at 20 °C. The sitting drop method was performed on 96-well MRC 2-drop crystallization plates in polystyrene (SWISSCI) containing 90 µl of crystallization solution in the reservoir.

### Crystallization of *Mt*ATPS

*Mt*ATPS (0.7 µl) at a concentration of 14 mg ml^−1^ (*Mt*ATPS form 1, Extended Data Table 1) or at a concentration of 27 mg ml^−1^ (*Mt*ATPS form 2) was mixed with 0.7 µl reservoir solution. *Mt*ATPS at 27 mg ml^−1^ was co-crystallized with 2 mM AMPcPP as well as 2 mM Na_2_SO_4_. For *Mt*ATPS form 1, transparent star-shaped crystals appeared after a few weeks in the following crystallization condition: 35% w/v pentaerythritol ethoxylate (15/4 EO/OH) and 100 mM 2-(*N*-morpholino)ethanesulfonic acid (MES) pH 6.5. For *Mt*ATPS form 2, transparent, long but thin plate-shaped crystals appeared after a few weeks in the following crystallization condition: 20% w/v polyethylene glycol 8000, 100 mM MES pH 6.0 and 200 mM calcium acetate.

### Crystallization of *Mt*APSK

*Mt*APSK (0.7 µl) at a concentration of 17.6 mg ml^−1^ was mixed with 0.7 µl reservoir solution and co-crystallized with 2 mM MgCl_2_. Transparent, plate-shaped crystals appeared after a few weeks in the following crystallization condition: 20% w/v polyethylene glycol 3350 and 100 mM tri-sodium citrate pH 5.5. *Mt*APSK was also crystallized with 2 mM MgCl_2_ and 2 mM APS but the obtained structures of those crystals were of lower resolution and without any substrate or product present in the active site.

### Crystallization of *Mt*PAPSR

*Mt*PAPSR (0.7 µl) at a concentration of 20 mg ml^−1^ was mixed with 0.7 µl reservoir solution and co-crystallized with FAD (0.5 mM final concentration). The crystal used for phasing was a brown flat square and appeared after a few days in the following crystallization condition: 40% v/v 2-methyl-2,4-pentanediol and 100 mM Tris/HCl pH 8.0.

The crystal used to refine at high resolution was brown with an elongated plate shape. It appeared after a few days in the following crystallization condition: 35% v/v 2-methyl-2,4-pentanediol, 100 mM Tris pH 7.0 and 200 mM NaCl. Before transfer to liquid N_2_, the crystal was soaked in 10 mM disodium 3’-phosphoadenosine 5’-phosphate for 7 min.

### Crystallization of *Mt*PAPP

*Mt*PAPP (0.7 µl) at a concentration of 20 mg ml^−1^ was mixed with 0.7 µl reservoir solution and co-crystallized with Tb-Xo4 (10 mM final concentration), MnCl_2_ (2 mM final concentration) and 2 mM PAP. The Tb-Xo4 is a nucleating/phasing agent^50^, which should increase the crystallization performance; however, in this case, the same crystalline form was obtained in the absence of the compound and diffracted to similar resolution. Transparent, bipyramid crystals appeared after a few weeks in the following crystallization condition: 1.6 M tri-sodium citrate.

### X-ray crystallography and structural analysis

*Mt*PAPSR crystal handling was done inside the Coy tent under anaerobic atmosphere (N_2_:H_2_, 97:3); the other crystals were handled under aerobic conditions. The crystals were directly plunged in liquid nitrogen or were soaked for 5–30 s in their crystallization solution supplemented with a cryoprotectant before being frozen in liquid nitrogen. For *Mt*ATPS form 2, 30% glycerol was used as cryoprotectant. For *Mt*APSK, 25% ethylene glycol was used as cryoprotectant.

Crystals were tested and collected at 100 K at different synchrotrons (Extended Data Table 1). Data were processed with autoPROC^51^ except for *Mt*PAPP, which gave better statistics with indexation by the X-ray Detector Software (XDS) and the scaling step performed with SCALA^52^. All data collection statistics are provided in Extended Data Table 1. *Mt*ATPS forms 1 and 2, *Mt*APSK and *Mt*PAPP were solved by using PHENIX with the following templates: 1V47 (ATPS from *T. thermophilus*) for *Mt*ATPS form 1, *Mt*ATPS form 1 for *Mt*ATPS form 2 and 5CB6 (APS kinase from *Synechocystis* sp.) for *Mt*APSK. For *Mt*PAPP, the template was created de novo using AlphaFold 2 (ref. ^32^).

For *Mt*PAPSR, an X-ray fluorescence spectrum on the Fe K-edge was measured to optimize the data collection at the appropriate wavelength. Datasets were collected at 1.73646 Å for the single-wavelength anomalous dispersion experiment. Native datasets were collected at a wavelength of 0.97625 Å on another crystal. Data were processed and scaled with autoPROC^51^. Phasing, density modification and automatic building were performed with CRANK-2 (ref. ^53^).

All models were manually rebuilt with COOT and further refined with PHENIX^54,55^. During the refinement, non-crystallographic symmetry and translational-libration screw were applied. For all structures except for ATPS form 1, hydrogens were added in riding position in the last refinement cycle. Hydrogens were removed in the final deposited models.

All models were validated using MolProbity^56^. Data collection and refinement statistics, as well as PDB identification codes for the deposited models and structure factors are listed in Extended Data Table 1. Figures were generated with PyMOL (Schrödinger). The metal in *Mt*ATPS was modelled as zinc using CheckMyMetal^57^.

### High-resolution clear native PAGE (hrCN PAGE)

To visualize the expression levels of *Mt*Fsr when cells were grown on different sulfur sources, hrCN PAGE was performed. *M. thermolithotrophicus* cultures (2 × 10 ml) were supplemented with either 2 mM Na_2_S, 2 mM Na_2_SO_3_, 2 mM Na_2_S and 2 mM Na_2_SO_4_, or 2 mM Na_2_SO_4_ as sulfur substrates and grown for one night at 65 °C, standing. Cells were collected by anaerobic centrifugation at 6,000 × *g* for 20 min at room temperature and the cell pellets were resuspended in 2 ml lysis buffer (50 mM tricine pH 8.0 and 2 mM sodium dithionite). The cells were sonicated 4× at 70% intensity for 10 s, followed by a 30 s break (MS 73 probe, SONOPULS Bandelin). The hrCN PAGE was run anaerobically and the protocol is detailed in Extended Data under hrCN PAGE preparation. One gel with an 8–15% acrylamide gradient was run (shown in Fig. 5b) and another one with a 5–15% acrylamide gradient (see Source Data Fig. 5).

### Coupled enzyme activity of *Mt*ATPS/*Mt*APSK

The activity of both enzymes was determined by the production of ADP which was coupled to NADH oxidation via pyruvate kinase and lactate dehydrogenase^58^. The assays were performed in a final volume of 100 µl 96-well deep-well plates and spectrophotometrically monitored (Omega multimode microplate reader) at 360 nm at 35 °C. KH_2_PO_4_ (100 mM) at pH 7.0, supplemented with 1.5 mM MgCl_2_ and 100 mM KCl, was used as a buffer. For NADH, a molar extinction coefficient of 4,546.7 cm^−1^ M^−1^ was experimentally determined for the above-named conditions. To the buffer, 1 mM NADH, 2.5 mM Na_2_SO_4_, 1 mM phosphoenolpyruvate (PEP), 2 mM ATP, 2 U inorganic pyrophosphatase (*Saccharomyces cerevisiae*, 10108987001, Sigma-Aldrich), 1.1 U ml^−1^ lactate dehydrogenase, 0.8 U ml^−1^ pyruvate kinase (rabbit muscle, P0294, Sigma-Aldrich) and 0.5 mg ml^−1^ *Mt*APSK (all final concentrations) were added. The reaction was started by the addition of 0.5 mg ml^−1^ *Mt*ATPS. Addition of 0.02 mM Na_2_MoO_4_ did not affect activity (0.116 ± 0.027 µmol of oxidized NADH min^−1^ mg^−1^), but the addition of 2 mM Na_2_MoO_4_ resulted in a decrease (0.068 ± 0.019 µmol of oxidized NADH min^−1^ mg^−1^). All assays were performed in triplicates.

### *Mt*PAPP enzyme assay

The activity of the *Mt*PAPP was determined by the production of orthophosphate, which was quantified using the malachite green phosphate assay kit (Sigma-Aldrich) by the formation of a green complex. The assays were performed in 96-well deep-well plates and the absorbance at 620 nm was spectrophotometrically followed (Omega multimode microplate reader). Tris/HCl (25 mM) at pH 7.64 was used as a buffer. Buffer, 40 µM PAP or 90 µM of AMP/ADP/ATP/APS or PP_i_, 1 mg ml^−1^ bovine serum albumin, 50 µM MnCl_2_ and/or 50 µM MgCl_2_ (final concentration) were mixed in a 1.5 ml Eppendorf tube on ice. Previously frozen *Mt*PAPP (0.5 µg ml^−1^ final concentration) was added and the mixture (final volume of 40 µl) was immediately incubated for 5 min at 40 °C. Next, 14 µl of the reaction mix was diluted in 66 µl of filtered Milli-Q H_2_O and immediately flash frozen in liquid N_2_ to quench the reaction. Then, 20 µl of malachite green reagent was added to the samples, the mixture was incubated at room temperature for 30 min and the formation of the green complex was measured at 620 nm. All assays were performed in triplicates. The measurements presented in Fig. 3a come from two different experiments (left and right subpanels). Both experiments were performed at two different days with the same enzyme preparation.

### Coupled *Mt*PAPSR assay

Since PAPS is unstable at high temperatures, we first tried to determine the activity of *Mt*PAPSR in the direction of PAPS production, as previously described for dissimilatory APS reductases for APS production^38^. PAPS oxidation was determined in 50 mM Tris/HCl buffer (pH 7.5) containing 5 mM Na_2_SO_3_, 2 mM PAP or 2 mM AMP (final concentrations) and 3.27 µg ml^−1^ *Mt*PAPSR. The reaction was started with a final concentration of 0.5 mM K_3_Fe(CN)_6_. The decrease in absorbance at 420 nm was measured and corrected for the background reaction without enzyme. No activity was detected. Therefore, we used the physiological reaction to monitor *Mt*PAPSR activity. To perform the coupled *Mt*PAPSR assay, the enzymes needed to be purified at the same time and immediately used for the assay (see Supplementary Materials for the detailed purification protocol for the enzymes used in this assay).

*Mt*PAPSR activity assays were carried out in an anaerobic atmosphere (100% N_2_) at 45 °C. The assays were performed in 200 µl final volume in 96-well deep-well plates and spectrophotometrically monitored on a SPECTROstar Nano microplate reader. HEPES (50 mM, pH 7.0) supplemented with 50 mM KCl, 1.5 mM MnCl_2_ and 1.5 mM MgCl_2_ was used as a buffer. Reduced methyl viologen (MV_red_, 0.5 mM) served as an electron donor for *Mt*PAPSR. The molar extinction coefficient (*ε*_600nm_ = 8,133.3 cm^−1^ M^−1^) was experimentally determined using the above-named conditions and by reducing methyl viologen with 2 mM sodium dithionite. For the assay, methyl viologen was reduced with carbon monoxide by the CO-dehydrogenase from *Clostridium autoethanogenum* according to a previously published protocol^59^. CO was exchanged for N_2_ and the MV_red_ was immediately used for the assay. To the buffer and MV_red_, 5 mM ATP, 1 mM sodium dithionite, 0.2 U pyrophosphatase (*E. coli*, MFCD00131379, Sigma-Aldrich), 0.127 mg ml^−1^ *Mt*ATPS, 0.12 mg ml^−1^ *Mt*APSK, 0.1 mg ml^−1^ *Mt*PAPP and 0.0645 mg ml^−1^ *Mt*PAPSR were added. The reaction was started by the addition of 5 mM Na_2_SO_4_ and followed by oxidation of MV_red_ at 600 nm. All assays were performed in triplicates.

### Sulfite reductase activity in cell extracts

To determine the sulfite reductase activity from *M. thermolithotrophicus*, cultures were grown on either 2 mM Na_2_S, 2 mM Na_2_SO_3_ or Na_2_SO_4_ in 10 ml of the above-mentioned medium in serum flasks. Cells (9 ml) were collected in late exponential phase (OD_600_: 3.45 for 2 mM Na_2_S, 3.91 for 2 mM Na_2_SO_3_, 3.37 for Na_2_SO_4_) by centrifugation at 6,000 × *g* for 10 min at 4 °C. The supernatant was discarded and the cell pellets were frozen in liquid N_2_. The pellets were then resuspended in 1 ml 0.5 M KH_2_PO_4_ pH 7.0. The cells were lysed by sonication (2× 10 s at 50% intensity, probe MS73, SONOPULS Bandelin), followed by centrifugation at 4 °C at 15,600 × *g*. The supernatant was passed through a 0.2 µm filter and the protein concentration was determined by the Bradford method (6.63 mg ml^−1^ for 2 mM Na_2_S, 6.14 mg ml^−1^ for 2 mM Na_2_SO_3_ and 6.31 mg ml^−1^ for Na_2_SO_4_). The activity assays were performed under an anaerobic atmosphere (100% N_2_) at 50 °C in 96-well deep-well plates and spectrophotometrically monitored (SPECTROstar Nano microplate reader). The assay mixture contained 0.5 M KH_2_PO_4_ pH 7.0, 118 µM MV_red_ (final concentration, previously reduced with the equimolar amount of sodium dithionite) and 30 µM Na_2_SO_3_ (final concentration). Under these conditions, a molar extinction coefficient of *ε*_600nm_ = 9,840 cm^−1^ M^−1^ was experimentally determined. The reaction was started by the addition of 0.05 µg of cell extract, followed by oxidation of MV_red_ at 600 nm. All assays were performed in triplicates.

### Phylogenetic trees

For a detailed description of the phylogenetic analysis, see Supplementary Materials^60^.

### Reporting summary

Further information on research design is available in the Nature Portfolio Reporting Summary linked to this article.

### Supplementary information

Supplementary Information Supplementary Figs. 1–8, Tables 1 and 2, Constructs and gene codon optimization, and Discussion.

Reporting Summary

Supplementary Materials Supplementary materials file.

Supplementary Data Statistical source data for Supplementary Fig. 2.

### Source data

Source Data Fig. 1 Statistical source data.

Source Data Fig. 2 Statistical source data.

Source Data Fig. 3 Statistical source data.

Source Data Fig. 4 Statistical source data.

Source Data Fig. 5 Statistical source data.

Source Data Fig. 5 Unprocessed native gels.

Source Data Fig. 6 Statistical source data.

Source Data Extended Data Fig. 2 Statistical source data.

Source Data Extended Data Fig. 8 Statistical source data.

## Figures and Tables

**Extended Data Table 1:**
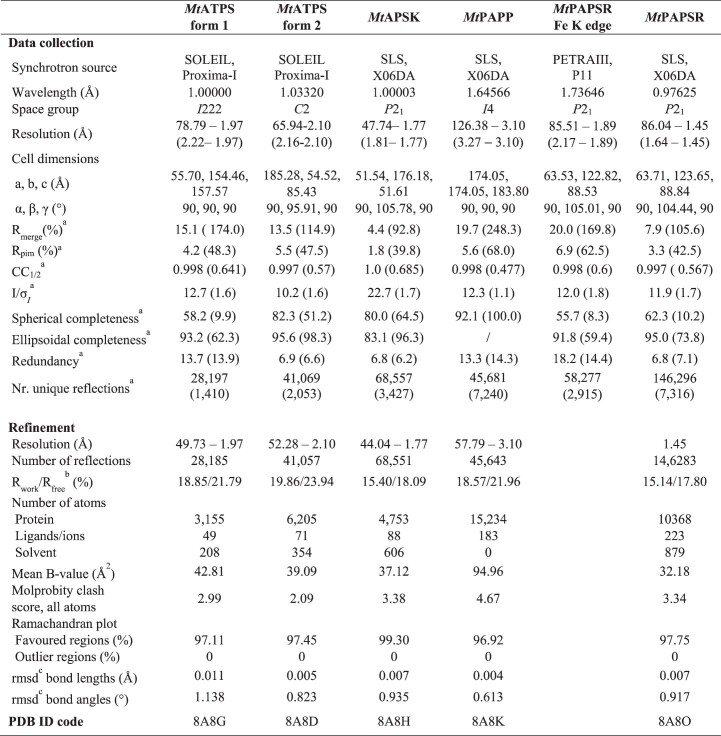
X-ray analysis statistics for the presented structures

^a^Values relative to the highest resolution shell are within parentheses.

^b^R_free_ was calculated as the R_work_ for 5 % of the reflections that were not included in the refinement.

^c^rmsd, root mean square deviation.

## Data Availability

All structures used for structural comparison are accessible from the Protein Data Bank and accordingly cited in the text. The structures were deposited in the Protein Data Bank under the ID: 8A8G for *Mt*ATPS form 1, 8A8D for *Mt*ATPS form 2, 8A8H for *Mt*APSK, 8A8K for *Mt*PAPP and 8A8O for *Mt*PAPSR. The data for this study are available in the paper and its Supplementary Information. Source data are provided with this paper.
