# Supplementary material for: Assimilatory sulfate reduction in the marine methanogen Methanothermococcus thermolithotrophicus
Source: Nat Microbiol. 2023 Jun 5;8(7):1227–39. doi: 10.1038/s41564-023-01398-8 (PMC10322725; doi:10.1038/s41564-023-01398-8)
Supplement: Supplementary file 1 — Supplementary Figs. 1–8, Tables 1 and 2, Constructs and gene codon optimization, and Discussion. [file 41564_2023_1398_MOESM1_ESM.pdf]

nature microbiology

Article

https://doi.org/10.1038/s41564-023-01398-8

Assimilatory sulfate reduction in the marine methanogen *Methanothermococcus thermolithotrophicus*

In the format provided by the authors and unedited

Table of contents.

1. Supplementary Fig. 1 Page 2
2. Supplementary Fig. 2 Page 3
3. Supplementary Fig. 3 Page 4
4. Supplementary Fig. 4 Page 5
5. Supplementary Fig. 5 Page 6
6. Supplementary Fig. 6 Page 7
7. Supplementary Fig. 7 Page 8
8. Supplementary Fig. 8 Page 9
9. Supplementary Table 1 Page 10
10. Supplementary Table 2 Page 11
12. Supplementary Constructs and gene codon optimisation Page 12-14
13. Supplementary Discussion Page 15-16

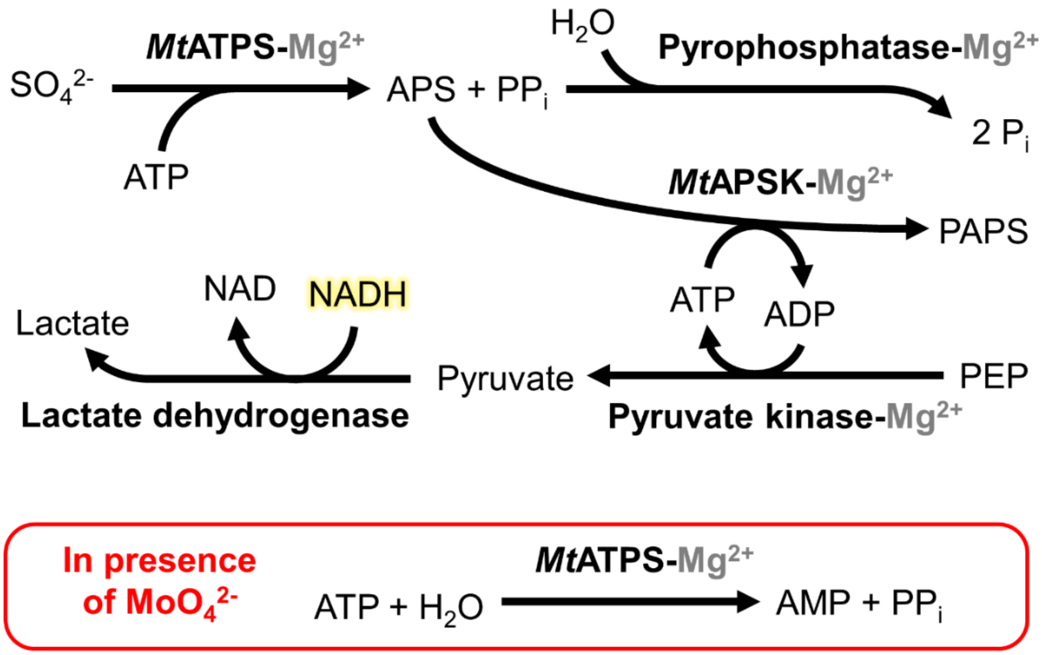

Supplementary Figure 1. Coupled enzyme assay and MoO_4^{2-} inhibition. Scheme of the coupled enzyme assay used in this study and impact of MoO_4^{2-} addition. When MoO_4^{2-} binds to the active site of the ATP-sulfurylase, ATP is hydrolysed into AMP and PP_i (molybdolysis).

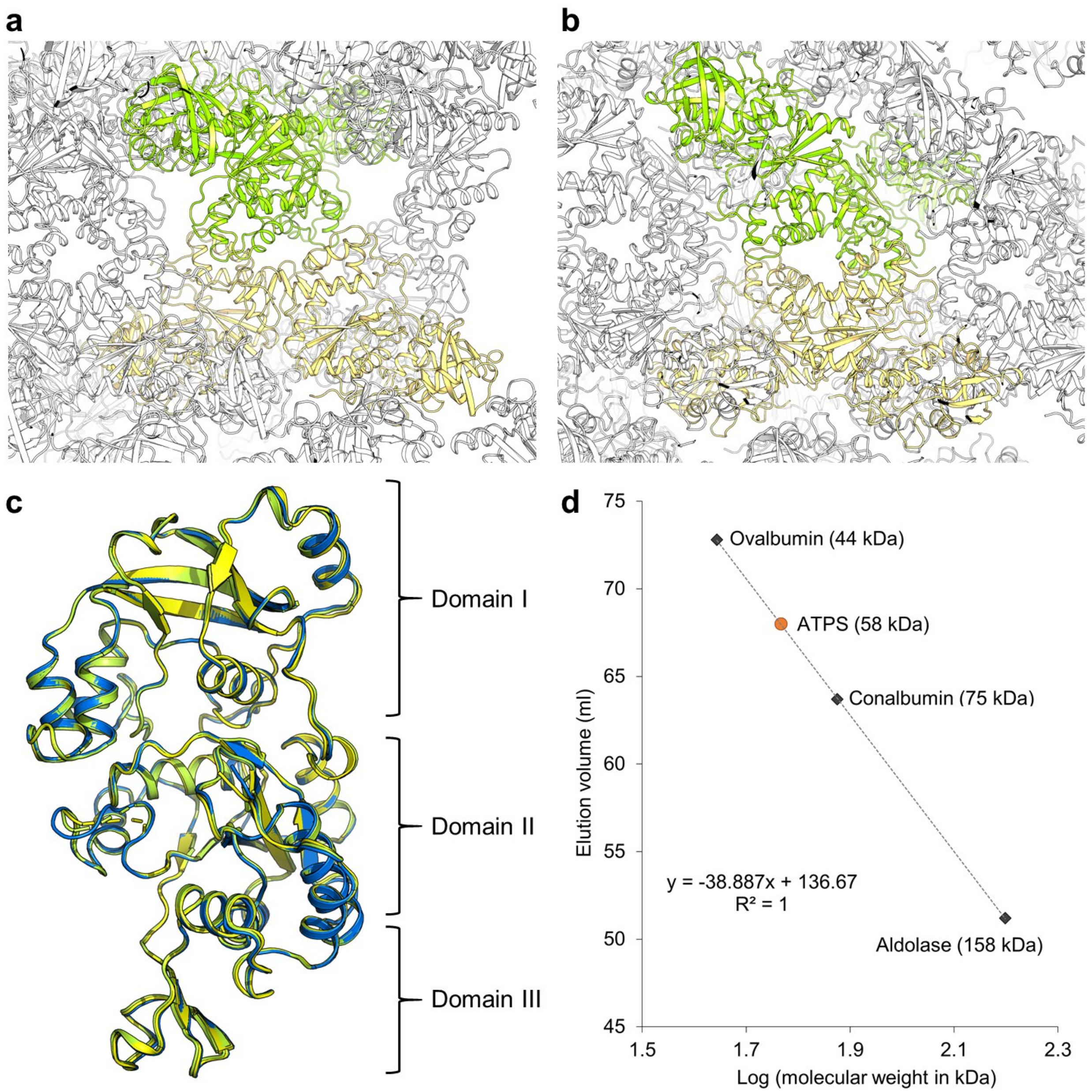

Supplementary Figure 2. Crystalline packing of *Mt*ATPS suggests a homotetramer. a, b, Packing of the crystalline form 1 (a) and 2 (b) containing a monomer and a dimer in the asymmetric unit, respectively. All *Mt*ATPS are shown as cartoons with the main dimeric unit coloured in light yellow. The opposite dimer, related by a 2-fold axis symmetry, is coloured in light green and suggest a tetrameric unit, contradicted by the PISA server and gel filtration (see panel d). **c**, The two monomers from *Mt*ATPS form 2 superpose well (rmsd 0.253 Å for 348 Cα, coloured in yellow and green) as well as the monomer from *Mt*ATPS form 1 (rmsd 0.238 Å for 331 Cα, coloured as blue). **d**, Gel filtration profile of *Mt*ATPS and a high molecular weight calibration kit (GE Healthcare). The obtained experimental molecular weight of 58 kDa is smaller than the expected dimeric form (89 kDa calculated from the sequence) but refutes a homotetrameric arrangement.

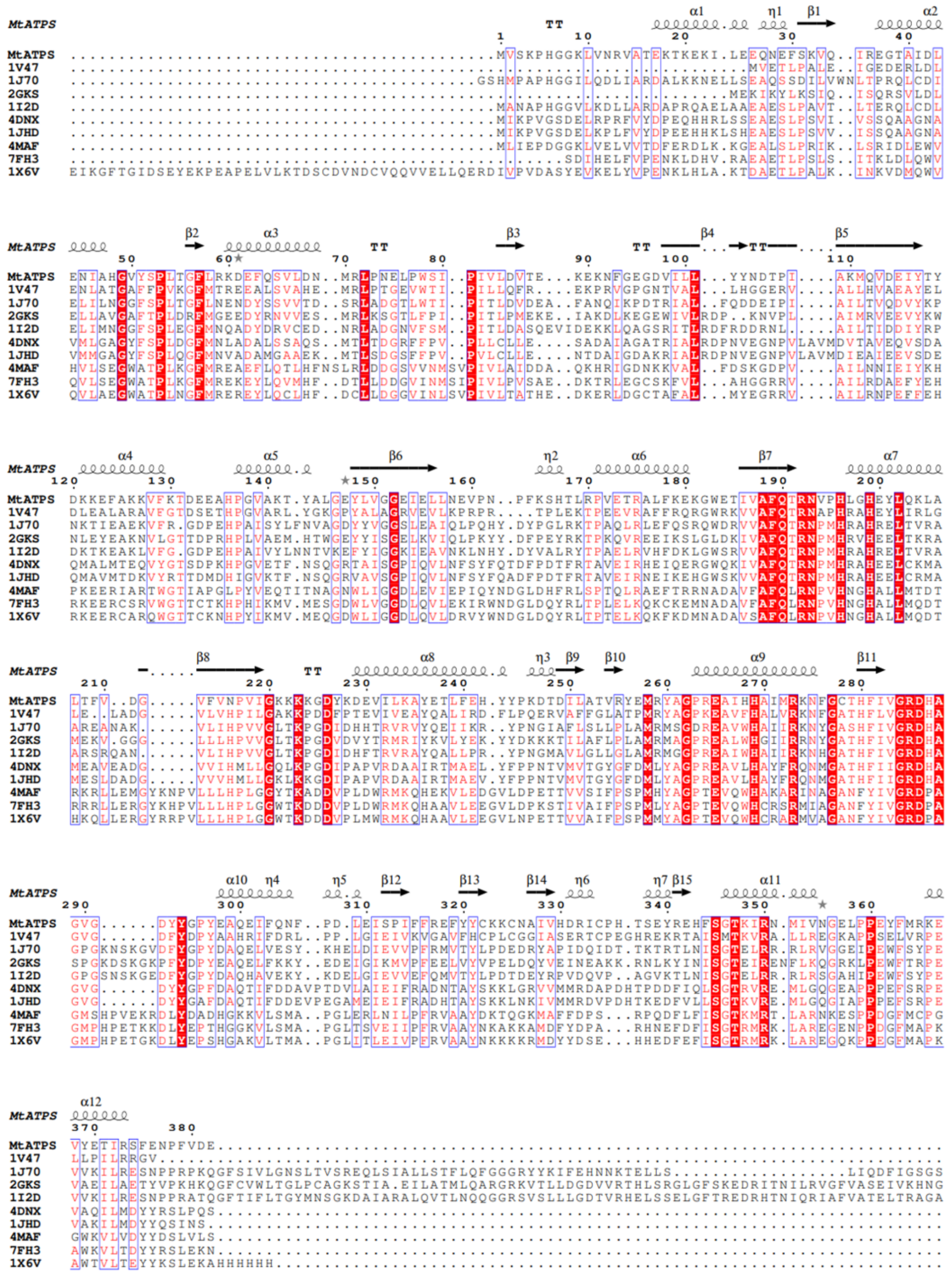

Supplementary Figure 3. Sequence conservation across *Mt*ATPS with homologues. Perfectly conserved residues are highlighted with a red background. Sequence alignment was done using MUSCLE[1], secondary structure prediction was performed with ESPript 3.0[2]. For more information regarding the PDB accession numbers, see Supplementary Table 1.

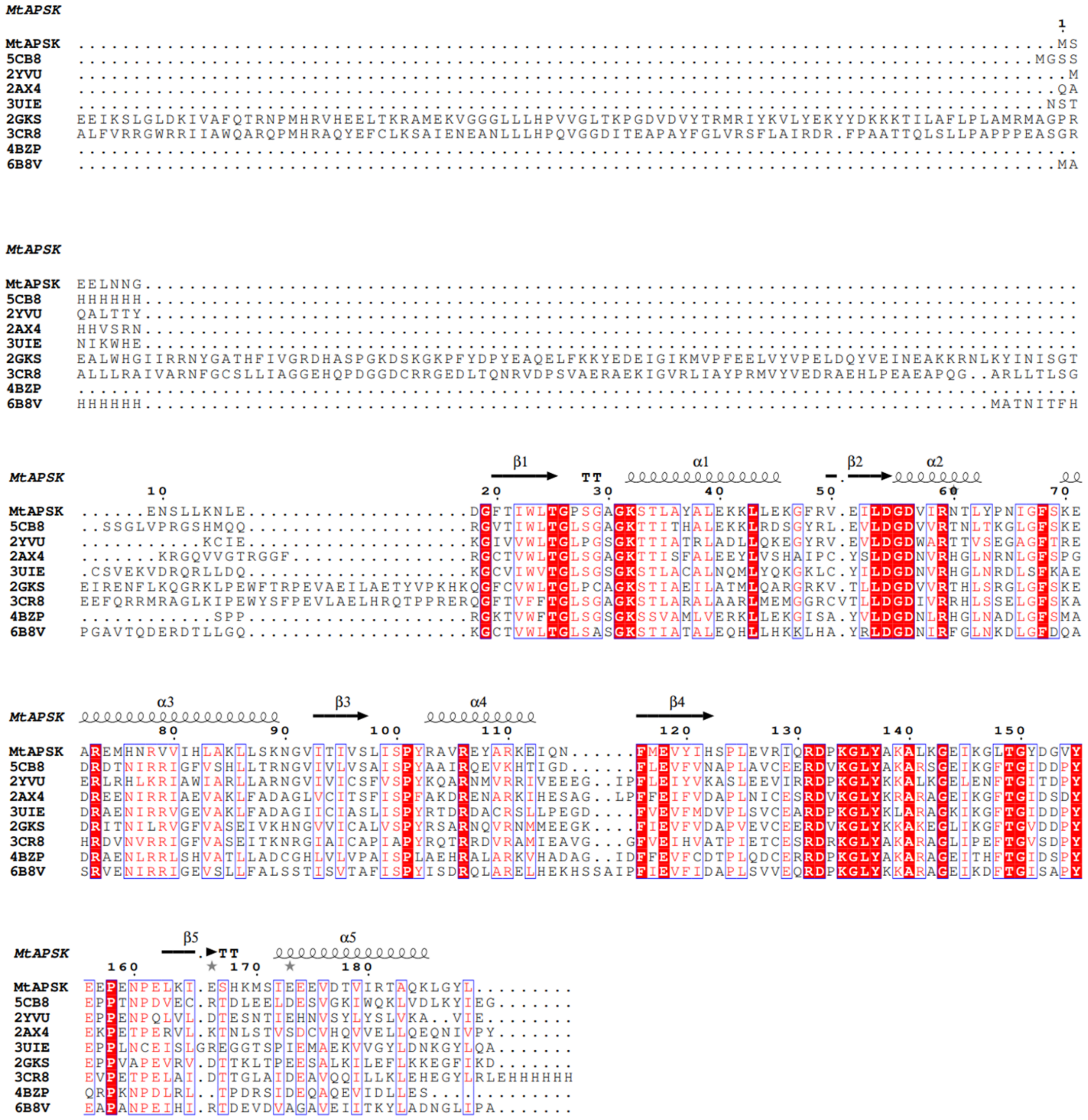

Supplementary Figure 4. Sequence conservation across *Mt*APSK with other APS-kinases. Perfectly conserved residues are highlighted with a red background. Sequence alignment was done using MUSCLE[1], secondary structure prediction was performed with ESPript 3.0[2]. For more information regarding the PDB accession numbers, see Supplementary Table 2.

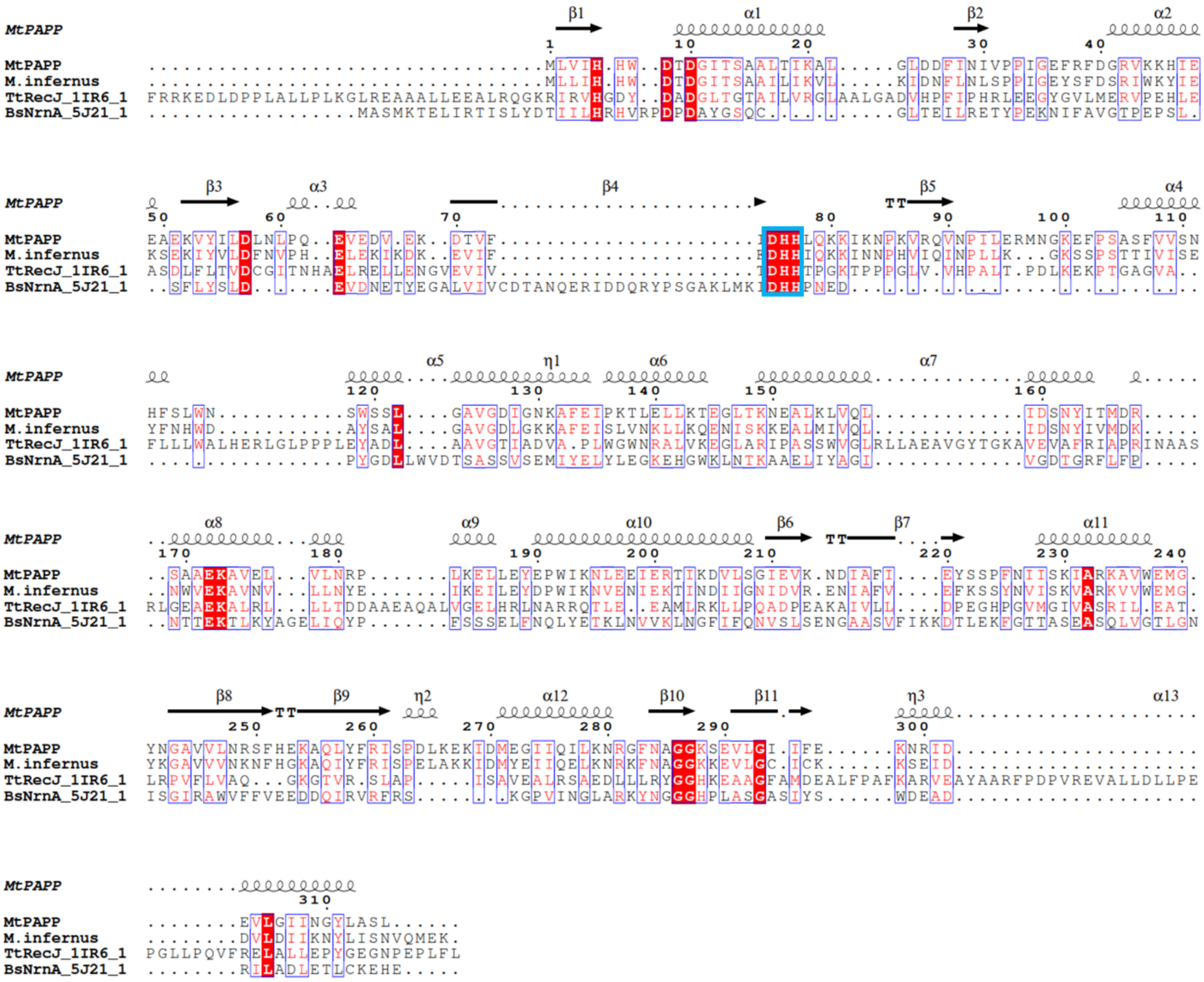

Supplementary Figure 5. Sequence conservation across *Mt*PAPP with the putative PAPP from *Methanocaldococcus infernus* and structural homologues. Perfectly conserved residues are highlighted with a red background and a blue box highlights the DHH motif. MtPAPP: *Methanothermococcus thermolithotrophicus* PAP-phosphatase; M.infernus: *Methanocaldococcus infernus* (WP_013099421, putative PAPP); TtRecJ: *Thermus thermophilus* exonuclease RecJ (PDB: 1IR6); BsNrnA_5J21: *Bacillus subtilis* bifunctional oligoribonuclease and PAP-phosphatase NrnA (PDB: 5J21). To allow a correct alignment, the "MGSSHHHHHHENLYFQS"-tag from BsNrnA_5J21 was removed. Sequence alignment was done using MUSCLE[1], secondary structure prediction was performed with ESPript 3.0[2].

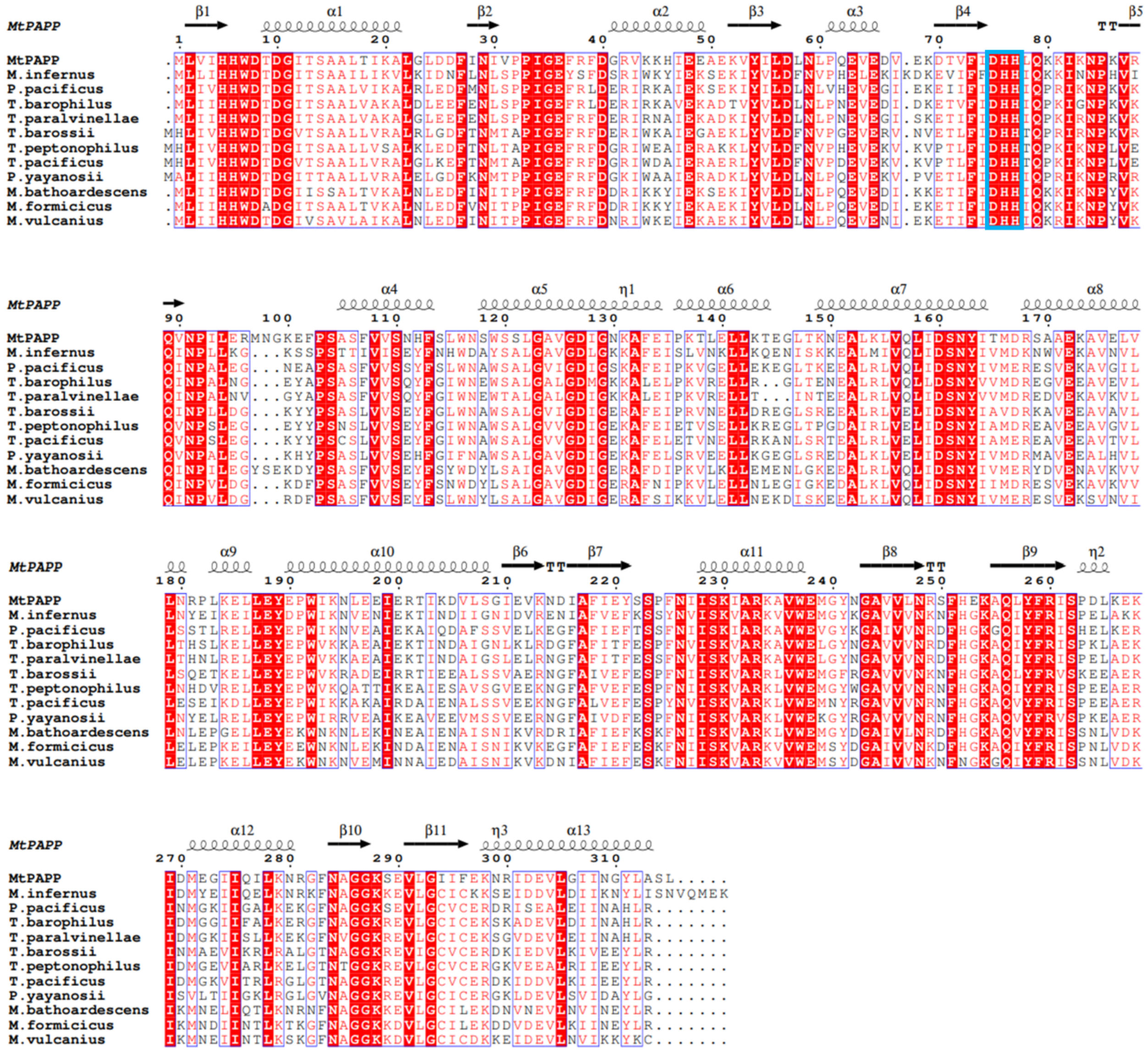

Supplementary Figure 6. Sequence alignment of *Mt*PAPP with archaeal homologues. Perfectly conserved residues are highlighted with a red background and a blue box highlights the DHH motif. MtPAPP: *Methanothermococcus thermolithotrophicus* PAP-phosphatase; M.infernus: *Methanocaldococcus infernus* (WP_013099421); P.pacificus: *Palaeococcus pacificus* (WP_048165810); T.barophilus: *Thermococcus barophilus* (WP_013468329); T.paralvinellae: *Thermococcus paralvinellae* (WP_042682556); T.barossii: *Thermococcus barossii* (WP_088865569); T.peptonophilus: *Thermococcus peptonophilus* (WP_062389597); T.pacificus: *Thermococcus pacificus* (WP_088853513); P.yayanosii: *Pyrococcus yayanosii* (WP_048058414); M.bathoardescens: *Methanocaldococcus bathoardescens* (WP_048201137); M.formicicus: *Methanotorris formicicus* (WP_007044583); M.vulcanius: *Methanocaldococcus vulcanius* (WP_012819478). Sequence alignment was done using MUSCLE[1], secondary structure prediction was performed with ESPript 3.0[2].

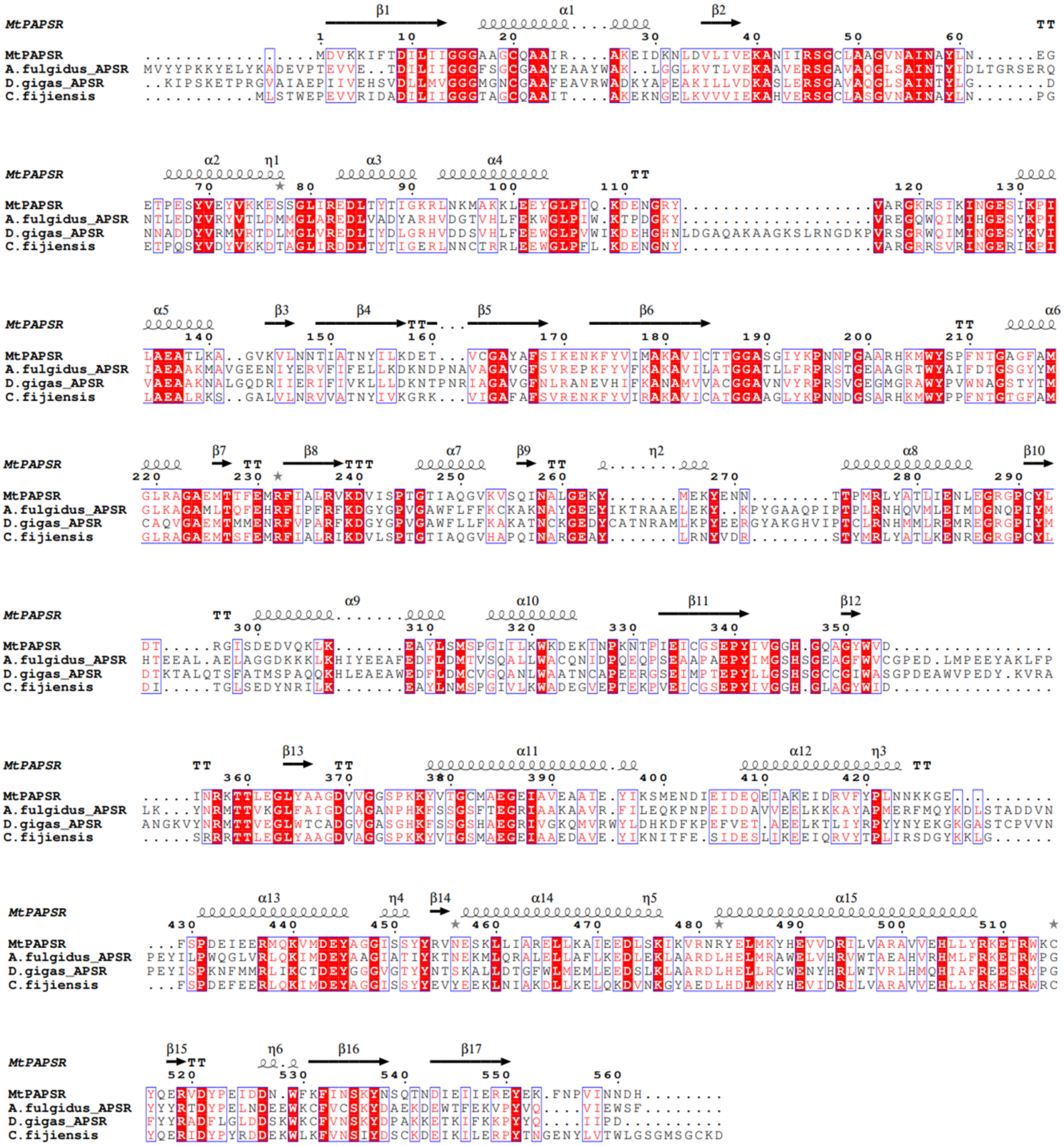

Supplementary Figure 7. Sequence alignment of dissimilatory APS-reductases with *Mt*PAPSR. Perfectly conserved residues are highlighted with a red background. MtPAPSR: PAPS-reductase from *Methanothermococcus thermolithotrophicus*, A.fulgidus_APSR: alpha subunit of the APSR from *Archaeoglobus fulgidus* (PDB: 2FJA), D.gigas_APSR: alpha subunit of the APSR from *Megalodesulfovibrio gigas* (PDB: 3GYX), and C.fijiensis: the putative APSR from *Caldanaerobius fijiensis* (*Cf*APSR, WP_073344903, closest homologue of *Mt*PAPSR alpha). Sequence alignment was done using MUSCLE[1], secondary structure prediction was performed with ESPript 3.0[2].

	ATP-sulfurylase (reference WP_018153795)	PAP-phosphatase (reference WP_018153796)	APS-kinase (reference WP_018153797)	PAPS-reductase alpha subunit (reference WP_018153799)
Methanopyrales	/	/	/	/
Methanococcales	*Methanotorris formicicus* (WP_048115642.1, 78 %)<br>*Methanocaldococcus bathoardescens* (WP_048201136.1, 79 %)<br>*Methanocaldococcus sp.* SG7 (WP_214399893.1; 73 %)<br>*Methanocaldococcus infernus* (WP_013099422.1; 71 %)<br>*Methanocaldococcus vulcanius* (WP_012819477.1; 77 %)<br>*Methanotorris formicicus* Mc-S-70 (EHP86153.1, 78 %)	*Methanotorris formicicus* (WP_007044583.1; 68 %)<br>*Methanocaldococcus bathoardescens* (WP_048201137.1; 67 %)<br>*Methanocaldococcus* sp. SG7 (WP_214399894.1, 62 %)<br>*Methanocaldococcus infernus* (WP_013099421.1; 60 %)<br>*Methanocaldococcus vulcanius* (WP_012819478.1; 64 %)	*Methanotorris formicicus* (WP_007044585.1; 76 %)<br>*Methanocaldococcus bathoardescens* (WP_048201139.1; 75 %)<br>*Methanocaldococcus sp. SG7* (WP_214399895.1, 76 %)<br>*Methanocaldococcus infernus* (WP_157198836.1; 75 %)<br>*Methanocaldococcus vulcanius* (WP_012819479.1; 76 %)	/
Methanobacteriales	/	/	/	/
Methanomicrobiales	*Methanoregulaceae* archaeon (NTV00908.1; 33 %)<br>*Methanomicrobiales* archaeon HGW-Methanomicrobiales-1 (PKL70343.1; 39 %)	/	*Methanoregulaceae archaeon* (RPI39683.1; 47 %)<br>*Methanomicrobiaceae archaeon* (MBN2735040.1; 49 %)<br>*Methanoregula formicica* (WP_015284456.1; 44 %)	/
Methanomassiliicoccales	/	/	/	/
Methanosarcinales	*Methanosarcinales* archaeon (RLG37318.1, 57 %)<br>*Methanosarcinales archaeon* (TRZ88670.1, 57 %)<br>*Candidatus* Methanoperedens sp. (NJD52436.1, 57 %)<br>*Methanohalophilus mahii* (WP_013037172.1; 55 %)<br>*Methanohalophilus* sp. RSK (WP_123135802.1; 54 %)<br>+ 23 more	*Methanosarcinales* archaeon (RLG38197.1; 28 %)<br>*Methanosarcinales* archaeon (MCD4846224.1, 32 %)<br>*Methanohalobium evestigatum* (WP_013194595.1; 33 %)	*Methanosarcinales* archaeon (RLG37639.1; 53 %)<br>*Methanosarcinales* archaeon (MCD4846223.1; 50 %)<br>*Methanohalobium evestigatum* (WP_013194596.1; 51 %)<br>*Methanococcoides orientis* (WP_233084240.1; 49 %)<br>*Methanohalophilus profundi* (WP_129597714.1; 48 %)<br>*Methanohalophilus portucalensis* (WP_072358461.1; 50 %)<br>+ 25 more	*Methanosarcinales* archaeon (RLG30695.1; 34 %)<br>*Methermicoccus shengliensis* (WP_052353262.1; 40 %)<br>*Candidatus* Methanoperedenaceae archaeon GB37 (CAD7771153.1; 36 %)<br>*Candidatus* Methanoperedenaceae archaeon GB50 (CAD7770084.1; 36 %)
Methanofastidiosa	/	/	/	/

Supplementary Figure 8. SO_4^{2-}-reduction associated genes across the seven orders of methanogens. The protein sequences from the biochemically and structurally characterized enzymes from *M. thermolithotrophicus* were used as reference. The NCBI accession numbers of the proteins (left) as well as the amino acid sequence identity (in %) in comparison to *M. thermolithotrophicus* enzymes are shown in brackets. Homologues below 28 % sequence identity or with a coverage below 85 % are not shown.

Supplementary Table 1. Sequence and structural alignment of *Mt*ATPS. Sequence alignment was performed using PyMOL version 2.2.0 (Schrödinger, LLC).

Name of the organisms	Abbreviation	PDB code	Alignment on Domain II Rmsd in Å (aligned Cα)	Overall alignment Rmsd in Å (aligned Cα)
Methanothermococcus thermolithotrophicus	*Mt*ATPS			
Thermus thermophilus	*Tt*ATPS	1V47	1.07 (121)	1.78 (296)
Saccharomyces cerevisiae	*Sc*ATPS	1J70	1.06 (112)	1.61 (266)
Aquifex aeolicus	*Aa*ATPS	2GKS	1.25 (125)	1.55 (272)
Penicillium chrysogenum	*Pc*ATPS	1I2D	1.12 (112)	1.54 (279)
Allochromatium vinosum	*Av*ATPS	4DNX	0.83 (116)	1.44 (257)
Riftia pachyptila symbiont	*Rrs*ATPS	1JHD	0.92 (124)	1.42 (260)
Glycine max	*Gm*ATPS	4MAF	1.27 (110)	2.33 (315)
Homo sapiens	*Hs*ATPS1	1X6V	1.25 (105)	1.98 (252)
Homo sapiens	*Hs*ATPS2	7FH3	1.10 (105)	1.83 (261)

Supplementary Table 2. Sequence and structural alignment of *Mt*APSK. Sequence alignment was performed using PyMOL version 2.2.0 (Schrödinger, LLC).

Name of the organisms	Abbreviation	PDB code	Overall alignment Rmsd in Å (aligned Cα)
Methanothermococcus thermolithotrophicus	*Mt*APSK		
Synechocystis sp. PCC 6803	*Ss*APSK	5CB6	1.33 (129)
Arabidopsis thaliana	*At*APSK	3UIE	1.59 (104)
Aeropyrum pernix	*Ap*APSK	2YVU	1.18 (117)
Penicillium chrysogenum	*Pc*APSK	1M7H	1.00 (120)
Homo sapiens	*Hs*APSK1	2OFW	1.13 (111)
Homo sapiens	*Hs*APSK2	2AX4	1.25 (123)
Aquifex aeolicus	*Aa*APSK	2GKS	1.77 (124)
Thiobacillus denitrificans	*Td*APSK	3CR8	1.14 (116)
Mycobacterium tuberculosis	*Mt*APSK	4BZP	1.13 (111)
Cryptococcus neoformans	*Cn*APSK	6B8V	0.81 (111)

Constructs and gene codon optimisation.

ATP-sulfurylase sequence from *M. thermolithotrophicus*

(NCBI Accession number: WP_018153795.1)

MVSKPHGGKLVNRVATEKTKEKILEEQNEFSKVQIREGTAIDLENIAHGVYSPLTGFLRKDEFQSVLDNMR
LPNELPWSIPIVLDVTEKEKNFGEGDVILLYYNDTPIAKMQVDEIYTYDKKEFAKKVFKTDEEAHPGVAKT
YALGEYLVGGEIELLNEVPNPFKSHTLRPVETRALFKEKGWETIVAFQTRNVPHLGHEYLQKLALTFVDGV
FVNPVIGKKKKGDYKDEVILKAYETLFEHYYPKDTDILATVRYEMRYAGPREAIHHAIMRKNFGCTHFIVG
RDHAGVGDYYGPYEAQEIFQNFPDLEISPIFFREFYYCKKCNAIVHDRICPHTSEYREHFSGTKIRNMIVNGE
LPPEYFMRKEVYETIRSFENPFVDE

Codon optimized *Mt*ATP-sulfurylase sequence cloned into pET-28a(+):

CATATGGTTAGCAAGCCGCACGGTGGCAAACTGGTGAATCGTGTGGCGACCGAGAAGACCAAGGAGA
AGATCCTGGAAGAACAGAACGAATTCAGCAAGGTGCAGATCCGTGAGGGTACCGCGATCGACCTGGA
AAACATTGCGCATGGTGTGTACAGCCCGCTGACCGGCTTCCTGCGTAAAGACGAGTTTCAAAGCGTTC
TGGATAACATGCGTCTGCCGAACGAACTGCCGTGGAGCATCCCGATTGTGCTGGATGTTACCGAGAAG
GAGAAGAACTTTGGCGAGGGCGACGTGATTCTGCTGTACTATAACGATACCCCGATCGCGAAGATGCA
GGTTGACGAGATTTACACCTATGATAAGAAAGAATTCGCGAAGAAAGTGTTTAAGACCGACGAGGAA
GCGCACCCGGGTGTTGCGAAAACCTACGCGCTGGGCGAGTATCTGGTGGGTGGCGAGATCGAACTGCT
GAACGAAGTTCCGAACCCGTTCAAGAGCCACACCCTGCGTCCGGTTGAAACCCGTGCGCTGTTCAAGG
AGAAAGGTTGGGAAACCATTGTGGCGTTTCAGACCCGTAACGTTCCGCACCTGGGTCACGAATACCTG
CAAAAACTGGCGCTGACCTTCGTGGATGGCGTGTTTGTTAACCCGGTTATCGGTAAGAAAAAGAAAGG
CGACTACAAGGATGAAGTGATTCTGAAAGCGTACGAAACCCTGTTCGAACACTACTATCCGAAGGACA
CCGATATCCTGGCGACCGTTCGTTACGAGATGCGTTATGCGGGTCCGCGTGAAGCGATCCACCATGCG
ATTATGCGTAAAAACTTCGGTTGCACCCACTTTATTGTGGGTCGTGACCACGCGGGTGTTGGTGATTAC
TATGGCCCGTATGAGGCGCAGGAAATTTTCCAAAACTTTCCGGACCTGGAGATCAGCCCGATTTTCTTT
CGTGAATTCTACTATTGCAAGAAATGCAACGCGATCGTGCACGATCGTATTTGCCCGCACACCAGCGA
GTACCGTGAACACTTTAGCGGTACCAAAATCCGTAACATGATTGTTAACGGCGAGCTGCCGCCGGAAT
ATTTTATGCGTAAGGAAGTTTATGAGACCATCCGTAGCTTTGAGAACCCGTTTGTTGATGAG**TGAGGA**
TCC

Restriction sites (NdeI and BamHI)

APS-kinase sequence from *M. thermolithotrophicus*

(NCBI Accession number: WP_018153797.1)

MSEELNNGENSLLKNLEDGFTIWLTGPSGAGKSTLAYALEKKLLEKGFRVEILDGDVIRNTLYPNIGFSKEA
REMHNRVVIHLAKLLSKNGVITIVSLISPYRAVREYARKEIQNFMEVYIHSPLEVRIQRDPKGLYAKALKGEI
KGLTGYDGVYEEPENPELKIESHKMSIEEEVDTVIRTAQKLGYL

Codon optimized *Mt*APS-kinase sequence cloned into pET-28a(+):

CATATGAGCGAGGAACTGAACAACGGCGAAAACAGCCTGCTGAAGAACCTGGAGGACGGCTTCACCA
TTTGGCTGACCGGTCCGAGCGGTGCGGGCAAGAGCACCCTGGCGTACGCGCTGGAAAAGAAACTGCT
GGAGAAAGGCTTCCGTGTGGAAATCCTGGACGGTGATGTTATTCGTAACACCCTGTATCCGAACATTG
GCTTTAGCAAGGAAGCGCGTGAGATGCACAACCGTGTGGTTATCCACCTGGCGAAGCTGCTGAGCAAA
AACGGTGTGATCACCATTGTTAGCCTGATCAGCCCGTACCGTGCGGTGCGTGAATATGCGCGTAAAGA
GATCCAGAACTTTATGGAAGTGTACATTCACAGCCCGCTGGAAGTGCGTATCCAACGTGACCCGAAGG
GCCTGTATGCGAAGGCGCTGAAAGGTGAAATTAAAGGTCTGACCGGCTACGATGGTGTTTATGAGGAA

CCGGAAAACCCGGAGCTGAAGATCGAGAGCCACAAAATGAGCATTGAGGAAGAGGTGGATACCGTTA
TCCGTACCGCGCAGAAACTGGGTTACCTG**TGA**GGATCC

Restriction sites (NdeI and BamHI)

PAP-phosphatase sequence from *M. thermolithotrophicus*

(NCBI Accession number: WP_018153796.1)

MLVIHHWDTDGITSAALTIKALGLDDFINIVPPIGEFRFDGRVKKHIEEAEKVYILDLNLPQEVEDVEKDTVF
IDHHLQKKIKNPKVRQVNPILERMNGKEFPSASFVVSNHFSLWNSWSSLGAVGDIGNKAFEIPKTLELLKTE
GLTKNEALKLVQLIDSNYITMDRSAAEKAVELVLNRPLKELLEYEPWIKNLEEIERTIKDVLSGIEVKNDIAF
IEYSSPFNIISKIARKAVWEMGYNGAVVLNRSFHEKAQLYFRISPDLKEKIDMEGIIQILKNRGFNAGGKSEV
LGIIFEKNR IDEVLGIINGYLASL

Codon optimized *Mt*PAPP sequence cloned into pET-28a(+):

CATATGCTGGTGATTCACCACTGGGACACCGATGGTATCACCAGCGCGGCGCTGACCATTAAAGCGCT
GGGTCTGGACGATTTCATCAACATTGTTCCGCCGATCGGCGAGTTCCGTTTTGACGGTCGTGTGAAGAA
ACACATCGAGGAAGCGGAAAAAGTTTACATTCTGGATCTGAACCTGCCGCAGGAAGTGGAAGACGTT
GAGAAGGATACCGTGTTTATCGACCACCACCTGCAGAAGAAAATTAAGAACCCGAAAGTGCGTCAAG
TTAACCCGATCCTGGAGCGTATGAACGGCAAAGAGTTCCCGAGCGCGAGCTTTGTGGTTAGCAACCAC
TTCAGCCTGTGGAACAGCTGGAGCAGCCTGGGTGCGGTGGGTGATATCGGTAACAAGGCGTTTGAGAT
TCCGAAAACCCTGGAGCTGCTGAAGACCGAAGGTCTGACCAAGAACGAAGCGCTGAAACTGGTTCAA
CTGATCGACAGCAACTACATTACGATGGACCGTAGCGCGGCGGAGAAGGCGGTGGAACTGGTTCTGA
ACCGTCCGCTGAAAGAGCTGCTGGAGTATGAACCGTGGATTAAGAACCTGGAGGAAATCGAACGTAC
CATTAAAGACGTGCTGAGCGGCATCGAGGTTAAGAACGATATCGCGTTCATTGAATACAGCAGCCCGT
TTAACATCATTAGCAAGATTGCGCGTAAAGCGGTTTGGGAGATGGGCTACAACGGTGCGGTGGTTCTG
AACCGTAGCTTCCACGAAAAAGCGCAGCTGTATTTTCGTATCAGCCCGGACCTGAAGGAGAAAATTGA
TATGGAAGGCATCATTCAAATCCTGAAAAACCGTGGTTTCAACGCGGGTGGCAAGAGCGAAGTGCTG
GGTATCATTTTTGAGAAGAACCGTATCGACGAAGTTCTGGGCATCATTAACGGTTATCTGGCGAGCCT
G**TGA**GGATCC

Restriction sites (NdeI and BamHI)

PAPS-reductase subunit sequences from *M. thermolithotrophicus*

PAPS-reductase subunit alpha sequence from *M. thermolithotrophicus*

(NCBI Accession number: WP_018153799.1)

MDVKKIFTDILIIGGGAAGCQAAIRAKEIDKNLDVLIVEKANIIRSGCLAAGVNAINAYLNEGETPESYVEYV
KKESSGLIREDLTYTIGKRLNKMAKKLEEYGLPIQKDENGRYVARGKRSIKINGESIKPILAEATLKAGVKV
LNNTIATNYILKDETVCGAYAFSIKENKFYVIMAKAVICTTGGASGIYKPNNPGAARHKMWYSPFNTGAGF
AMGLRAGAEMTTFEMRFIALRVKDVISPTGTIAQGVKVSQINALGEKYMEKYENNTTPMRLYATLIENLEG
RGPCYLDTRGISDEDVQKLKEAYLSMSPGIILKWKDEKINPKNTPIEICGSEPYIVGGHGQAGYWVDINRKT
TLEGLYAAGDVVGGSPKKYVTGCMAEGEIAVEAAIEYIKSMENDIEIDEQEIAKEIDRVFYPLNNKKGEFSP
DEIEERMQKVMDEYAGGISSYYRVNESKLLIARELLKAIEEDLSKIKVRNRYELMKYHEVVDRILVARAVV
EHLLYRKETRWKCYQERVDYPEIDDNWFKFINSKYNSQTNDIEIIEREYEKFNPVINNDH

PAPS-reductase subunit beta sequence from *M. thermolithotrophicus*

(NCBI Accession number: WP_018153800.1)

MTIRIIEEICIGCGLCTKVCPGNLLYQREDGKSEIMDKRDCWDCAACVKECPVNAIEMYLQPEIGGRGSTLK
AKKTDDSIVWIITDNNGEEEVIEVKNKKTFDM

Codon optimized PAPS-reductase sequences cloned into pET-28a(+):

CCATGG***ATG***GATGTTAAGAAGATATTCACAGATATACTCATAATAGGTGGTGGTGCAGCAGGTTGCCA
GGCAGCAATAAGGGCAAAGGAGATAGATAAGAACCTCGATGTTCTCATAGTTGAGAAGGCAAACATA
ATAAGGTCAGGTTGCCTCGCAGCAGGTGTTAACGCAATAAACGCATACCTCAACGAGGGTGAGACAC
CCGAGTCATACGTTGAGTACGTTAAGAAGGAGTCATCAGGTCTCATAAGGGAGGATCTCACATACACA
ATAGGTAAGAGGCTCAACAAGATGGCAAAGAAGCTCGAGGAGTACGGTCTCCCCATACAGAAGGATG
AGAACGGTAGGTACGTTGCAAGGGGTAAGAGGTCAATAAAGATAAACGGTGAGTCAATAAAGCCCAT
ACTCGCAGAGGCAACACTCAAGGCAGGTGTTAAGGTTCTCAACAACACAATAGCAACAAACTACATA
CTCAAGGATGAGACAGTTTGCGGTGCATACGCATTCTCAATAAAGGAGAACAAGTTCTACGTTATAAT
GGCAAAGGCAGTTATATGCACAACAGGTGGTGCATCAGGTATATACAAGCCCAACAACCCCGGTGCA
GCAAGGCACAAGATGTGGTACTCACCCTTCAACACAGGTGCAGGTTTCGCAATGGGTCTCAGGGCAGG
TGCAGAGATGACAACATTCGAGATGAGGTTCATAGCACTCAGGGTTAAGGATGTTATATCACCCACAG
GTACAATAGCACAGGGTGTTAAGGTTTCACAGATAAACGCACTCGGTGAGAAGTACATGGAGAAGTA
CGAGAACAACACAACACCCATGAGGCTCTACGCAACACTCATAGAGAACCTCGAGGGTAGGGGTCCC
TGCTACCTCGATACAAGGGGTATATCAGATGAGGATGTTCAGAAGCTCAAGGAGGCATACCTCTCAAT
GTCACCCGGTATAATACTCAAGTGGAAGGATGAGAAGATAAACCCCAAGAACACACCCATAGAGATA
TGCGGTTCAGAGCCCTACATAGTTGGTGGTCACGGTCAGGCAGGTTACTGGGTTGATATAAACAGGAA
GACAACACTCGAGGGTCTCTACGCAGCAGGTGATGTTGTTGGTGGTTCACCCAAGAAGTACGTTACAG
GTTGCATGGCAGAGGGTGAGATAGCAGTTGAGGCAGCAATAGAGTACATAAAGTCAATGGAGAACGA
TATAGAGATAGATGAGCAGGAGATAGCAAAGGAGATAGATAGGGTTTTCTACCCCCTCAACAACAAG
AAGGGTGAGTTCTCACCCGATGAGATAGAGGAGAGGATGCAGAAGGTTATGGATGAGTACGCAGGTG
GTATATCATCATACTACAGGGTTAACGAGTCAAAGCTCCTCATAGCAAGGGAGCTCCTCAAGGCAATA
GAGGAGGATCTCTCAAAGATAAAGGTTAGGAACAGGTACGAGCTCATGAAGTACCACGAGGTTGTTG
ATAGGATACTCGTTGCAAGGGCAGTTGTTGAGCACCTCCTCTACAGGAAGGAGACAAGGTGGAAGTG
CTACCAGGAGAGGGTTGATTACCCCGAGATAGATGATAACTGGTTCAAGTTCATAAACTCAAAGTACA
ACTCACAGACAAACGATATAGAGATAATAGAGAGGGAGTACGAGAAGTTCAACCCCGTTATAAACAA
CGATCAC**AGCAGCGGCCACCACCACCACCACCACTGA**GCTAGCATGACTGGTGGACAGCAAATGGG
TCGCGAAGGAGATATACC***ATG***ACAATAAGGATAATAGAGGAGATATGCATAGGTTGCGGTCTCTGCAC
AAAGGTTTGCCCCGGTAACCTCCTCTACCAGAGGGAGGATGGTAAGTCAGAGATAATGGATAAGAGG
GATTGCTGGGATTGCGCAGCATGCGTTAAGGAGTGCCCCGTTAACGCAATAGAGATGTACCTCCAGCC
CGAGATAGGTGGTAGGGGTTCAACACTCAAGGCAAAGAAGACAGATGATTCAATAGTTTGGATAATA
ACAGATAACAACGGTGAGGAGGAGGTTATAGAGGTTAAGAACAAGAAGACATTCGATATG**TGAGGA**
TCC

Red = Insertion of an internal RBS, Blue=linker, **Restriction sites (NcoI and BamHI)**

Supplementary Discussion.

Enzymatic rates.

The specific activities for the coupled enzymes ATPS-APSK are low but within the range described for other organisms (*i.e. Zea mays* ATPS has a specific enzyme activity of 0.000246 µmol/min/mg; *Arabidopsis thaliana* ATPS has a specific enzyme activity of ~0.145 µmol/min/mg; *Saccharomyces cerevisiae* ATPS has a specific enzyme activity of 0.69-140 µmol/min/mg according to the Brenda database).

*Mt*ATPS, *Mt*APSK and *Mt*PAPSR are assimilating enzymes. Therefore, their turnover may not be as high as dissimilatory ones. They may even be tightly regulated (*i.e.* competitive inhibition by end products, as shown by the retro-inhibition of PAP in the absence of PAPP) to avoid excessive cellular energy consumption.

We cannot exclude that our experimental set-up had a negative effect on the enzymatic rates, as we could not use high salt concentrations (high KPO_4^{2-} concentrations have been shown to increase enzymatic activity for certain hydrogenotrophic methanogens[3], but cannot be used here as PO_4^{2-} is a reaction product that would disturb the assay equilibrium), nor could we work at physiological temperature (65 °C) because we used enzymes from mesophiles (*i.e.* pyrophosphatase from *E. coli*) in the coupled assays. In addition, the artificial electron donor methyl viologen is a surrogate, and the physiological electron donor could significantly favour the reaction towards sulfite production, thus triggering the equilibrium.

Origin of the enzymes constituting the SO_4^{2-}-assimilatory pathway.

Our structural and phylogenetic analyses suggest that *Mt*ATPS and *Mt*APSK are closely related to the thermophilic gram-negative bacterium *Thermus thermophilus* and marine archaeon *Aeropyrum pernix*, respectively. Since ATPS and APSK coding genes have only been found in less than 40 methanogens (Supplementary Fig. 8), it would argue for a lateral gene transfer rather than an ancestral origin common to all methanogens followed by the loss of these genes[4]. Retrieving the original donors to the different methanogen species would require deeper analyses and a larger set of sequences.

The tree suggests that *Mt*PAPSR is of bacterial origin and was probably acquired by horizontal gene transfer from a bacterium (possibly from *Thermincola potens* or within its clade).

The archaeal PAP phosphatases form their own clade. Once more, only a limited number of methanogen genomes harbour a PAPP coding gene (Supplementary Fig. 8). Therefore, the genes might have been horizontally transferred from an archaeon (e.g. belonging to the *Thermococcales*). Since archaeal PAP-phosphatases belong to the DHH family, we propose that this new class probably evolved from an ancestor containing the DHH motif.

It has been proposed that methanogenesis and sulfate reduction may have been intertwined pathways for more than 3.4 Gyr.[5] Sulfate assimilation might have been more prevalent in ancient methanogens, and while most of them lost the ability to assimilate SO_4^{2-}, some may have retained the required enzymes and adapted them to serve a hitherto unknown sulfur trafficking function. The pathway presented in our work would require a set of genes that only a few methanogenic genomes encode. If sulfate reduction was used in ancient methanogens, then it is unlikely that it was the one described in *M. thermolithotrophicus*.

Why would *M. thermolithotrophicus* assimilate SO_4^{2-}?

The archaeon was collected in geothermally heated sediments in an active volcanic area close to the shore of Naples. If the environment mimics a volcanic area, there could be soluble iron (Fe^{2+}) that will react with the sulfide and, with that, deplete it as bioavailable sulfur. While other methanogens have been shown to use metal sulfides as a source of iron and sulfur, this still needs to be clarified for *M. thermolithotrophicus*. Volcanic sulfate aerosols or marine sulfate ions are an alternative sulfur source. As shown in our results, a sulfate concentration in the range of 100 μM would be enough to sustain robust sulfur assimilation that should be available in this niche. Future *in situ* work and meta-transcriptomics on samples from this site would provide additional clues if *M. thermolithotrophicus* is indeed fixing sulfate in its native environment.

References

1 Edgar, R. C. MUSCLE: a multiple sequence alignment method with reduced time and space complexity. *BMC Bioinformatics* **5**, 113, doi:10.1186/1471-2105-5-113 (2004).

2 Robert, X. & Gouet, P. Deciphering key features in protein structures with the new ENDscript server. *Nucleic Acids Res* **42**, W320-W324, doi:10.1093/nar/gku316 (2014).

3 Setzke, E., Hedderich, R., Heiden, S. & Thauer, R. K. H_2: heterodisulfide oxidoreductase complex from *Methanobacterium thermoautotrophicum*. **220**, 139-148, doi:doi.org/10.1111/j.1432-1033.1994.tb18608.x (1994).

4 Fuchsman, C. A., Collins, R. E., Rocap, G. & Brazelton, W. J. J. P. Effect of the environment on horizontal gene transfer between bacteria and archaea. **5** (2017).

5 Susanti, D. & Mukhopadhyay, B. An intertwined evolutionary history of methanogenic archaea and sulfate reduction. *PLoS One* **7**, doi:10.1371/journal.pone.0045313 (2012).
