# Supplementary material for: Assimilatory sulfate reduction in the marine methanogen Methanothermococcus thermolithotrophicus
Source: Nat Microbiol. 2023 Jun 5;8(7):1227–39. doi: 10.1038/s41564-023-01398-8 (PMC10322725; doi:10.1038/s41564-023-01398-8)
Supplement: Supplementary file 3 — Supplementary materials file. [file 41564_2023_1398_MOESM3_ESM.pdf]

Supplementary materials file

Assimilatory sulfate-reduction in the marine methanogen *Methanothermococcus thermolithotrophicus*

Marion Jespersen[1] and Tristan Wagner[1]*

[1] Microbial Metabolism research group, Max Planck Institute for Marine Microbiology, Bremen, Germany. Correspondence: twagner@mpi-bremen.de

Extended materials and methods.

All plots presented in the study were generated with Microsoft Excel 16 (16.05356.1000).

All microbial cultures were spectrophotometrically monitored on a Spectrophotometer UV/VIS UVmini-1240 SHIMADZU.

Sulfur-free cultivation medium for *Methanococcales*. Per liter of medium: 558 mg of KH_2PO_4 (final concentration 4.1 mM), 1 g of KCl (13.4 mM), 25.13 g of NaCl (430 mM), 840 mg of $NaHCO_3$ (10 mM), 367.5 mg of $CaCl_2 \cdot 2\ H_2O$ (2.5 mM), 7.725 g of $MgCl_2 \cdot 6\ H_2O$ (38 mM), 1.18 g of NH_4Cl (22.06 mM), 61.16 mg of nitrilotriacetic acid (0.32 mM), 6.16 mg of $FeCl_2 \cdot 4\ H_2O$ (0.031 mM), 10 µl of 2 mM $Na_2SeO_3 \cdot 5\ H_2O$ stock (0.02 µM), 3.3 mg of $Na_2WO_4 \cdot 2\ H_2O$ (0.01 mM) and 2.42 mg of $Na_2MoO_4 \cdot 2\ H_2O$ (0.01 mM) were dissolved under constant stirring with 750 ml of deionized H_2O (dH_2O). 1 ml of 1.5 mM Resazurin and 10 ml of sulfur-free trace elements (see below) were subsequently added. In flasks the pH was set to either 7.6 with 50 mM Tris/HCl as buffer or to 6.2 with 50 mM 2-(*N*-morpholino)ethanesulfonic acid (MES). For the fermenter, 10 mM MES pH 6.2 was used as a buffer. The medium was filled up to a final volume of 1 L by the addition of dH_2O.

The cultivation media were transferred in a 1 L pressure protected DURAN® laboratory bottle with a magnetic stirring bar. The Duran flask was closed with a butyl rubber stopper and degassed by applying 3 min of vacuum, followed by 30 seconds addition of 1 X 10^5 Pa N_2:CO_2 atmosphere (90:10), under constant magnetic stirring. This was repeated for a minimum of 15 cycles and at the final gas addition step, an overpressure of 0.3 x 10^5 Pa N_2:CO_2 was applied.

Trace element composition for *Methanococcales*. A 100-fold-concentrated trace element solution was prepared by first dissolving 1.36 g of nitrilotriacetic acid (7.1 mM) in 800 ml dH_2O under magnetic stirring. The pH was shifted to 6.2 by adding NaOH pellets. 89.06 mg of $MnCl_2 \cdot 4\ H_2O$ (0.45 mM), 183.3 mg of $FeCl_3 \cdot 6\ H_2O$ (0.68 mM), 60.27 mg of $CaCl_2 \cdot 2\ H_2O$ (0.41 mM), 180.8 mg of $CoCl_2 \cdot 6\ H_2O$ (0.76 mM), 90 mg of $ZnCl_2$ (0.66 mM), 37.64 mg of $CuCl_2$ (0.28 mM), 46

mg of $Na_2MoO_4 \cdot 2\ H_2O$ (0.19 mM), 90 mg of $NiCl_2 \cdot 6\ H_2O$ (0.38 mM) and 30 mg of VCl_3 (0.19 mM) was added separately. The trace element mixture was filled up to a final volume of 1 L with dH_2O.

Media for *Archaeoglobus fulgidus*. The media were modified from DSMZ media 399. It was prepared in a Widdel flask and contains per liter of dH_2O: 0.14 g of KH_2PO_4, 0.25 g of NH_4Cl, 18 g of NaCl, 3.45 g of $MgSO_4 \cdot 7\ H_2O$, 4 g of $MgCl_2 \cdot 6\ H_2O$, 0.34 g of KCl, 0.14 g of $CaCl_2 \cdot 2\ H_2O$ and 1 ml of $Fe(NH_4)_2(SO_4)_2 \cdot 6\ H_2O$ (1.91 $mg.ml^{-1}$). The media was autoclaved for 25 minutes at 121 °C. Afterwards, the solution was transferred in a 1 L Duran bottle. 1 X 10^3 Pa $N_2:CO_2$ (90:10) overpressure was applied during the addition of 1 ml of the trace elements M141 (see below), 0.05 mg of Vitamin B_{12} (sterile filtered), 1 ml of a Se/Wo-solution (400 mg NaOH, 8 mg of $Na_2WO_4 \cdot 2\ H_2O$, 6 mg of $Na_2SeO_3 \cdot 5\ H_2O$ were solved in 1 L dH_2O and autoclaved at 121 °C for 25 minutes), 1 ml of riboflavin (17.5 mM of acetic acid, 2.5 mg of riboflavin 5`-monophosphate sodium salt dihydrate were dissolved in 100 ml dH_2O, sterile filtered and stored in the dark at 4 °C), 0.1 ml of a thiamine solution (for a 100 ml solution 10 mg thiamine chloride hydrochloride were dissolved in 50 mM Na_2HPO_4/H_3PO_4 pH 3.7, sterile filtered and stored in autoclaved brown flasks at 4 °C until usage), 1 ml of 1 $mg.ml^{-1}$ Resazurin, 30 ml of 1 M $NaHCO_3$, and 1 ml of 5-vitamin mix (see below). For the Na_2S grown cultures, 2 ml of 1 M Na_2S and some crystals of sodium dithionite (until colour loss) were added but omitted for the Na_2SO_4-grown culture. The pH was adjusted to 6.9 using 2 M HCl.

Trace element composition for *Archaeoglobus fulgidus*. The 10 x trace element solution was modified from DSMZ media 141. 1.5 g nitrilotriacetic acid were dissolved in 80 ml Milli-Q® H_2O, and the pH was set to 6.5 using 1 M KOH. Subsequently 3 g of $MgSO_4 \cdot 7\ H_2O$, 0.5 g of $MnSO_4 \cdot H_2O$, 1 g of NaCl, 100 mg of $FeSO_4 \cdot 7\ H_2O$, 152 mg of $CoCl_2 \cdot 6\ H_2O$, 100 mg of $CaCl_2 \cdot 2\ H_2O$, 180 mg of $ZnSO_4 \cdot 7\ H_2O$, 10 mg of $CuSO_4 \cdot 5\ H_2O$, 20 mg of $KAl(SO_4)_2 \cdot 12\ H_2O$, 10 mg of H_3BO_3, 10 mg of $Na_2MoO_4 \cdot 2\ H_2O$ and 30 mg of $NiCl_2 \cdot 6\ H_2O$ were added and a pH 7.0 was set with 1 M KOH and the solution was filled up to 100 ml with Milli-Q® H_2O. The trace elements were autoclaved at 121 °C for 25 minutes and then stored at room temperature in the dark.

Archaeoglobus fulgidus 5 - Vitamin mix. 15 mg pyridoxine hydrochloride, 10 mg nicotinic acid, 5 mg calcium-D(+)-pantothenate, 4 mg 4-aminobenzoic acid, and 1 mg D(+)-biotin were dissolved in 100 ml 10 mM Na_2HPO_4 at pH 7.1, sterile filtered and stored at 4 °C until usage.

Protein overexpression. The *Mt*ATPS, *Mt*APSK and *Mt*PAPP constructs expressed in *Escherichia coli* strain BL21(DE3) were cultivated in 1 to 3 L of Lysogeny Broth (per liter of medium: 10 g tryptone, 5 g yeast extract, 10 g NaCl) supplemented with a final concentration of 50 µg/ml kanamycin. Cultures were incubated by shaking at 220 rotation per minute (rpm) at 37 °C until an OD_{600nm} of 0.6 – 0.8 was reached. Induction was performed by adding a final concentration of 0.75 mM Isopropyl ß-D-1-thiogalactopyranoside (IPTG), and the cells were incubated for another hour by shaking at 37 °C. Cells were harvested by centrifugation at 5,000 × *g* for 20 min at 21 °C. Cell pellets were frozen in liquid N_2 and stored at -80 °C until further use.

For overexpression of the *Mt*PAPSR construct, *E. coli* BL21(DE3) was previously transformed with the plasmid pDB1282. The transformed cells were grown in a fermenter at 34 °C containing 8 L of modified Terrific Broth medium. For 8 L medium 96 g tryptone, 112 g yeast extract, 40 g glycerol, 4 g ferric ammonium citrate, 21 g MOPS pH 7.4 were dissolved in 7.2 L dH_2O and autoclaved. Then 800 ml TB salts (10 x; 18.5 g KH_2PO_4, 100.32 g K_2PO_4, autoclaved) supplemented with glucose (28 mM final, sterile filtered), kanamycin (50 µg/ml final, sterile filtered), ampicillin (100 µg/ml final, sterile filtered) and riboflavin (20 µg/ml final), were added. The preculture was grown in classic Terrific Broth medium. The cells were gassed with a constant flow of 15 x 10^4 Pa compressed air until they reached an OD_{600nm} of 1.74, then the gas was switched to a constant flow of 15 x 10^4 Pa N_2, to establish anaerobic conditions. Next, the cells were induced with a final concentration of 0.2 % L-arabinose, 25 mM sodium fumarate dibasic, 2 mM cysteine hydrochloride and 50 µM IPTG followed by incubation for one hour at 28 °C. The cells were harvested under anaerobic conditions (N_2:CO_2 with a 90:10 ratio) by centrifugation at 5,000 × *g* for 20 min at 4 °C. Cell pellets were frozen in liquid N_2 and stored at -80 °C until further use.

*Mt*ATPS, *Mt*APSK and *Mt*PAPP tag-cleavage and purification. 6 g, 9 g and 7 g (wet weight) of respectively *Mt*ATPS, *Mt*APSK, *Mt*PAPP-overexpressed *E.coli* cells were thawed under warm water and were resuspended in 22-50 ml lysis buffer (50 mM Na_2HPO_4 pH 8.0, 500 mM NaCl, 20 mM Imidazole, 5 % Glycerol) on ice. The cell lysate was homogenized by sonication: 10 cycles with 1 min at 80 % intensity followed by 1.5 min break (probe KE76, SONOPULS Bandelin) and cell debris were removed via centrifugation (45,000 x *g*, 50 min at 4 °C). The filtered sample was applied to a 5 ml HisTrap high performance column (GE healthcare, Germany), which was previously equilibrated with lysis buffer. The column was then washed with 2 column volumes of lysis buffer. A gradient of 0.02 to 0.3 M Imidazole was applied for 40 min at a flow rate of 1.5 $ml.min^{-1}$ and fractions of 1 ml were collected. *Mt*ATPS eluted between 0.1 and 0.15 M Imidazole, *Mt*APSK between 0.12 and 0.2 M and *Mt*PAPP eluted between 0.13 and 0.22 M Imidazole. The protein fractions were pooled and the buffer was exchanged for Phosphate buffer saline (137 mM NaCl, 2.7 mM KCl, 10 mM Na_2HPO_4, 1.8 mM KH_2PO_4 pH 7.4) by using 30-kDa-cutoff filter (6 ml, Merck Millipore, Darmstadt, Germany) for *Mt*ATPS and *Mt*APSK and a 10-kDa-cuttoff filter for *Mt*PAPP. The proteins were concentrated to 3 ml for the *Mt*PAPP and 5 ml for the *Mt*ATPS and *Mt*APSK. As the protein aggregated over time, the *Mt*PAPP containing the His-Tag was immediately passed onto a Superdex 200 Increase 10/300 GL (GE Healthcare), equilibrated in the following buffer 25 mM Tris/HCl pH 7.6, containing 5 % v/v glycerol and 2 mM dithiothreitol. *Mt*PAPP eluted at a flow rate of 0.8 $ml.min^{-1}$ in a sharp Gaussian peak at an elution volume of 75 ml. The fractions of interest containing *Mt*PAPP were concentrated with a 10 kDa cut-off centrifugal concentrator to 150 µl, exchanged with the storage buffer (25 mM Tris/HCl pH 7.6, containing 150 mM NaCl, 10 % v/v glycerol and 2 mM dithiothreitol), and was directly used for crystallization. The concentration of purified *Mt*PAPP, estimated by the Bradford method, was 20 $mg.ml^{-1}$.

For the *Mt*ATPS and *Mt*APSK, 50 µl of 0.1 $U.mg^{-1}$ Thrombin (from bovine plasma, Sigma-Aldrich, Germany) was added to the 5 ml of protein and incubated overnight at 22 °C to remove

the His-Tag of the enzymes. The samples were then passed onto a HiLoad® 16/600 Superdex® 200 pg (GE Healthcare), equilibrated in storage buffer (25 mM Tris/HCl pH 7.6, containing 150 mM NaCl, 10 % v/v glycerol and 2 mM dithiothreitol). *Mt*ATPS and *Mt*APSK eluted at a flow rate of 0.8 ml.min^{-1} in a sharp Gaussian peak at an elution volume of 68 ml and 81 ml, respectively. The fractions of interest containing the proteins were concentrated with a 30-kDa cut-off centrifugal concentrator (6 ml, Merck Millipore, Darmstadt, Germany) to 150 µl and the proteins were immediately used for crystallization. The concentration of purified *Mt*ATPS, estimated by the Bradford method, was 27 mg.ml^{-1} and 17.6 mg.ml^{-1} for *Mt*APSK.

*Mt*PAPS-reductase purification. 26 g (wet weight) of *Mt*PAPSR-overexpressed *E.coli* cells from the fermenter were thawed under warm water and transferred to an anaerobic tent containing an atmosphere of N_2:CO_2 (with a 90:10 ratio). 120 ml lysis buffer (50 mM Na_2HPO_4, 500 mM NaCl, 20 mM Imidazole, and 5 % v/v Glycerol) was added and cells were lysed by sonication: 5 cycles with 1 min at 75 % intensity followed by 3 min break (probe KE76, SONOPULS Bandelin). Cell debris were removed anaerobically via centrifugation (45,000 x *g*, 45 min at 4 °C). The supernatant was transferred to a Coy tent (N_2:H_2 atmosphere with a 97:3 ratio) under yellow light at 20 °C and filtered through a 0.2 µm filter (Sartorius). The filtered sample was applied to a 5 ml HisTrap high performance column (GE healthcare), which was previously equilibrated with lysis buffer. The column was then washed with 2 column volumes of lysis buffer. A gradient of 0.02 to 0.3 M Imidazole was applied for 40 min at a flow rate of 1.5 ml.min^{-1} and fractions of 1 ml were collected. *Mt*PAPSR eluted between 0.04 and 0.15 M Imidazole. The fractions of interest were merged and diluted with 4 volumes of 50 mM Tricine/NaOH pH 8.0 and 2 mM dithiothreitol. The sample was filtered through 0.2 µm and was loaded on a 5 ml Q Sepharose high performance column (GE healthcare). A gradient of 0 to 0.55 M NaCl was applied for 90 min with a flow rate of 1 ml.min^{-1}. Fractions of 1.5 ml were collected. *Mt*PAPSR eluted between 0.11 and 0.52 M NaCl.

The purest *Mt*PAPSR fractions were pooled and the buffer was exchanged for storage buffer (25 mM Tris/HCl pH 7.6, containing 10 % v/v glycerol and 2 mM dithiothreitol (DTT)) by using 30 kDa cut-off filter (6 ml, Merck Millipore, Darmstadt, Germany) and *Mt*PAPSR was concentrated to 400 µl. The concentrated sample was passed onto a Superdex 200 Increase 10/300 GL (GE Healthcare), equilibrated in storage buffer. *Mt*PAPSR eluted at a flow rate 0.4 ml.min^{-1} in a sharp Gaussian peak at an elution volume of 12.5 ml. The fractions of interest containing *Mt*PAPSR were concentrated with a 30 kDa cut-off centrifugal concentrator to 300 µl and the protein was directly used for crystallization. For the activity assays *Mt*PAPSR was incubated with 0.5 mM FAD for 15 min to promote cofactor integrity, followed by buffer exchange to remove excess FAD using a 30 kDa cut-off concentrator. The concentration of purified *Mt*PAPSR, estimated by the Bradford method, was 20 mg.ml^{-1}.

Enzyme purification for coupled *Mt*PAPSR assay. For each step, the *Mt*ATPS, *Mt*APSK and *Mt*PAPP were handled under aerobic conditions and on ice, while the *Mt*PAPSR was always kept in an anaerobic atmosphere (N_2:CO_2 with a 90:10 ratio) at room temperature. To save time, the

His-tags were not cleaved off. We previously saw that the tag did not interfere with the activity of these enzymes.

5.4 g, 7.6 g, 6.3 g and 11.0 g (wet weight) of recombinantly expressed *Mt*ATPS, *Mt*APSK, *Mt*PAPSR and *Mt*PAPP cells (as described above), respectively, were resuspended in 30 ml lysis buffer (50 mM Na_2HPO_4, pH 8.0, 500 mM NaCl, 20 mM Imidazole, 5 % Glycerol), separately. The cells were broken by the following sonication protocol: 5 cycles with 30 seconds at 75 % intensity followed by 1.5 min break (probe KE76, SONOPULS Bandelin) and cell debris were removed anaerobically via centrifugation (45,000 x *g*, 45 min at 4 °C). The filtered supernatant was applied on a Ni-NTA gravity column (1 ml Ni-NTA resin equilibrated with lysis buffer). The column was washed with 8 ml lysis buffer, then the proteins were eluted by applying 3 ml elution buffer (50 mM Na_2HPO_4, pH 8.0, 500 mM NaCl, 300 mM Imidazole, 5 % Glycerol). The flow trough was collected, filtered and injected onto a Superdex 200 Increase 10/300 GL (GE Healthcare), equilibrated in storage buffer (25 mM Tris/HCl pH 7.6, containing 10 % v/v glycerol and 2 mM DTT). The fractions containing the sample of interest were pooled and concentrated using 10 kDa (*Mt*PAPP) and 30 kDa cut-off centrifugal concentrator (Sartorius). The yield of this purification was: 2 ml of *Mt*ATPS at 11 $mg.ml^{-1}$, 1.5 ml of *Mt*APSK at 8 $mg.ml^{-1}$, 2 ml of the *Mt*PAPP at 3 $mg.ml^{-1}$ and 2 ml of the *Mt*PAPSR at 4.5 $mg.ml^{-1}$.

<u>High resolution Clear Native PAGE preparation.</u> The whole process was performed anaerobically in an anoxic chamber (N_2:CO_2 with a 90:10 ratio). Anaerobic fresh or frozen samples were used. Glycerol (20% v/v final) was added to each sample and 0.001% w/v <u>Ponceau S</u> serves as a marker for protein migration. The electrophoresis cathode buffer contained 50 mM Tricine; 15 mM Bis-Tris, pH 7; 0.05% w/v sodium deoxycholate; 0.01% w/v dodecyl <u>maltoside</u> and 2 mM of DTT. The anode buffer contained 50 mM Bis-Tris buffer pH 7 and 2 mM DTT. High resolution Clear Native PAGE were carried out using a 5 to 15% linear polyacrylamide gradient and gels were run with a constant 40 mA current (PowerPac™ Basic Power Supply, Bio-Rad).

<u>Phylogenetic trees</u>. Phylogenetic analyses were performed using MEGA11.0 by applying default parameters.[60] Homolog proteins were first identified and obtained from NCBI using BLASTP (E-value cut-off of 1e1). The protein sequences were then aligned using MUSCLE and all homologs were identified through an iterative alignment evaluation based on characterized proteins and manual filtering. The evolutionary history was inferred using the Neighbor-Joining method. The bootstrap consensus tree inferred from 2000 replicates is taken to represent the evolutionary history of the taxa analysed. Branches corresponding to partitions reproduced in less than 50 % bootstrap replicates are collapsed. The percentage of replicate trees in which the associated taxa clustered together in the bootstrap test (2000 replicates) are shown next to the branches. The evolutionary distances were computed using the JTT matrix-based method and are in the units of the number of amino acid substitutions per site.
