# Supplementary figures and images for: Assimilatory sulfate reduction in the marine methanogen Methanothermococcus thermolithotrophicus
Source: Nat Microbiol. 2023 Jun 5;8(7):1227–39. doi: 10.1038/s41564-023-01398-8 (PMC10322725; doi:10.1038/s41564-023-01398-8)

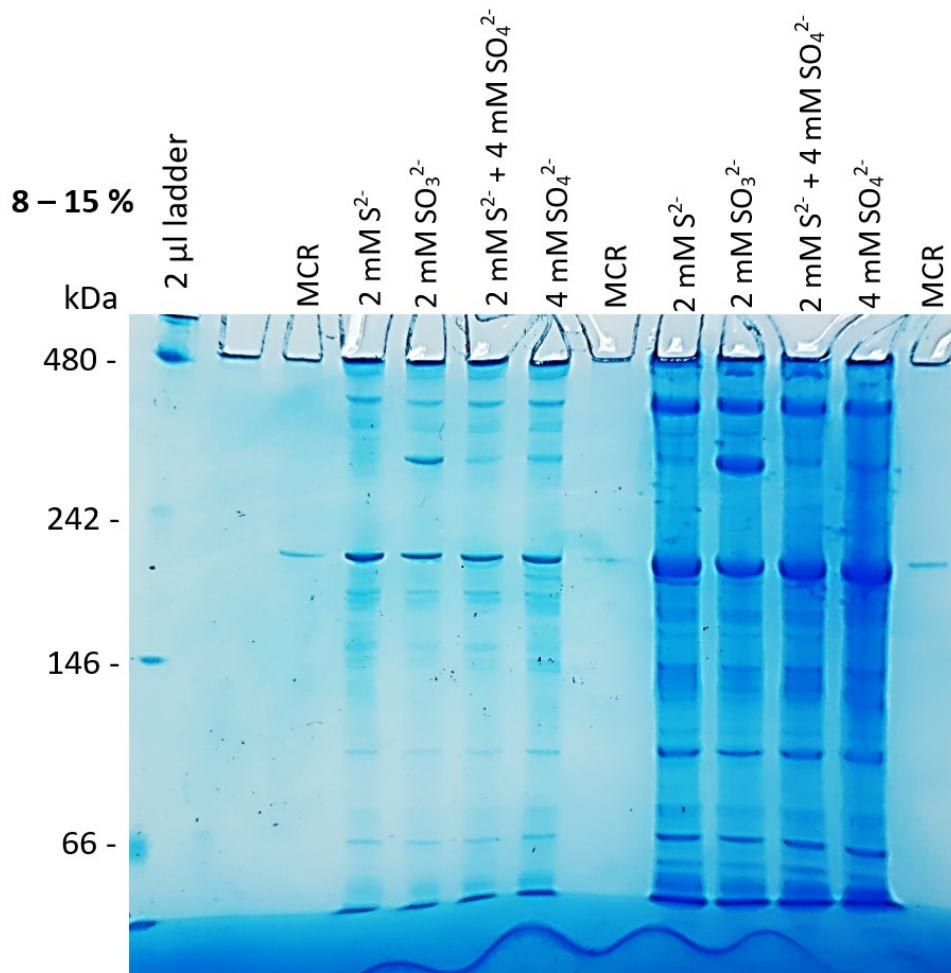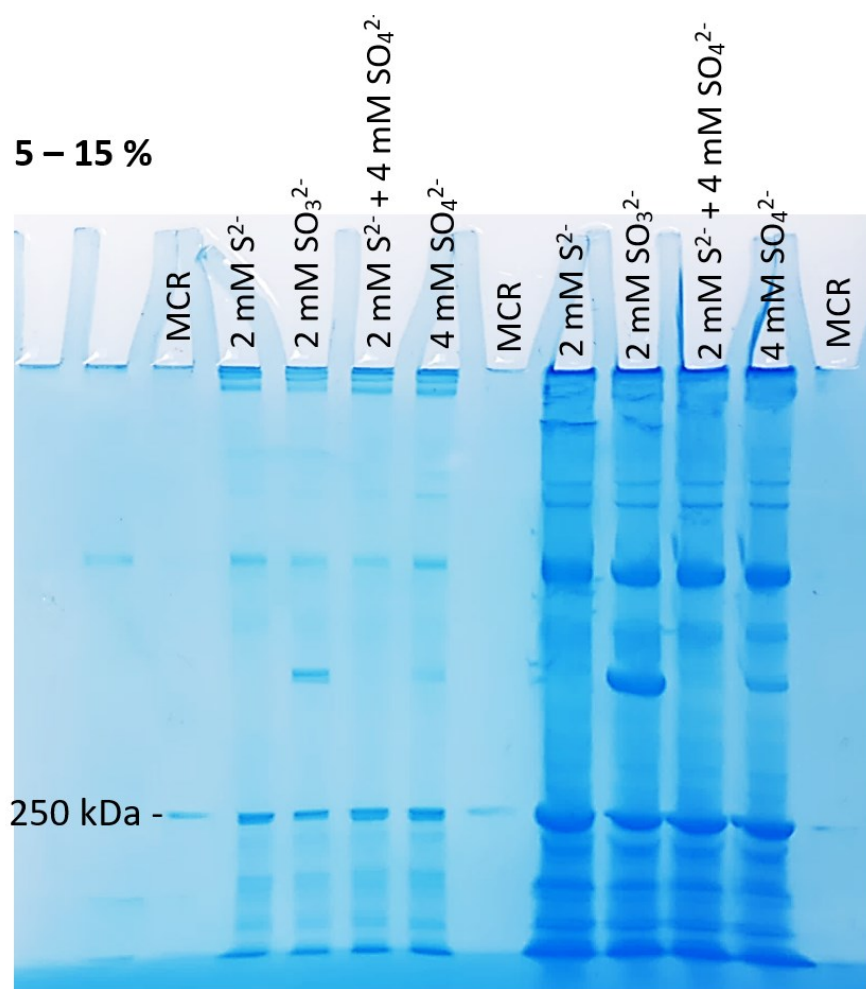

Supplement: Supplementary file 10 — Unprocessed native gels. [file 41564_2023_1398_MOESM10_ESM.pdf]
